# Epidermal Patch Technologies for Integrated Healthcare and Infection Management

**DOI:** 10.1002/adhm.71189

**Published:** 2026-04-27

**Authors:** Yuqi Wang, Atakan Tevlek, Pawel L. Urban, Boaz Mizrahi, Onome Ejeromedoghene, Roshan Deen, Yuanjing Lin, Jagan Mohan Dodda

**Affiliations:** ^1^ School of Microelectronics Southern University of Science and Technology Shenzhen China; ^2^ Department of Medical Biology Faculty of Medicine Atilim University Ankara Turkey; ^3^ Department of Chemistry National Tsing Hua University Hsinchu Taiwan; ^4^ Israel Institute of Technology Faculty of Biotechnology and Food Engineering Haifa Israel; ^5^ State and Local Joint Engineering Laboratory For Novel Functional Polymeric Materials College of Chemistry Chemical Engineering and Materials Science Soochow University Suzhou Jiangsu Province P. R. China; ^6^ Materials For Medicine Research Group School of Medicine The Royal College of Surgeons in Ireland (RCSI) Medical University of Bahrain Busaiteen Kingdom of Bahrain; ^7^ New Technologies – Research Centre (NTC) University of West Bohemia Pilsen Czech Republic

**Keywords:** biomedical applications, epidermal patches, hydrogel‐based devices, wearable electronics

## Abstract

Epidermal patches are multifunctional skin‐interfacing platforms with applications spanning wound management, real‐time biosensing, drug delivery, and tissue regeneration. Hydrogels play a central role due to their mechanical compliance, water‐rich composition, and tunable physicochemical properties. Key design features flexibility, stretchability, self‐healing, and self‐adhesion, which ensure stable skin contact and device stability. Tailored electrical conductivity, enabled by conductive polymers, fillers, and novel fabrication strategies, allows seamless integration with bioelectronics for intelligent health monitoring. Fabrication innovations, such as 3D/4D printing, stereolithography, digital light processing, extrusion‐based writing, inkjet printing, electrospinning, and microneedle‐based platforms, allow precise spatial control and multifunctional integration. Emerging approaches, including AI‐assisted biosensing, stimuli‐responsive drug release, noninvasive skin metabolite monitoring, and biodegradable systems, further expand their potential. Applications range from infection‐resistant wound dressings and minimally invasive drug delivery to acne therapy, cardiac patches, and hydrogel micropatch probes for skin metabolomics. Challenges remain in achieving scalable manufacturing, long‐term durability, and material sustainability. Future development will converge intelligent hydrogel design, integrated biosensing, data‐driven analytics, advanced metabolomics, and personalized transdermal therapeutic, transforming epidermal patches from passive materials into adaptive, closed‐loop biointerfaces capable of sensing, decision‐making, and on‐demand intervention. By uniting therapeutic, diagnostic, and protective functions, hydrogel‐based epidermal patches are set to revolutionize personalized healthcare.

## Graphical abstract

Epidermal patches have evolved from simple wound coverings into multifunctional, skin‐conformable platforms integrating drug delivery, biosensing, and therapeutic functionalities. This review highlights their material innovations, fabrication strategies, and intelligent designs, including hydrogels, microneedles, and flexible electronics, while emphasizing emerging AI‐driven, self‐healing, and adaptive systems that collectively enable next‐generation, closed‐loop personalized healthcare solutions.

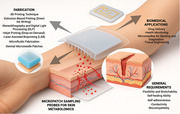

## 1 Introduction

Epidermal patches are thin, flexible, and often skin‐conformable platforms that have evolved from simple wound coverings into multifunctional biomedical interfaces integrating drug delivery, biosensing, and therapeutic functionalities [1]. Initially used as protective physical barriers for wounds, they have undergone substantial evolution over the past decades [2]. While their historical roots trace back to ancient civilizations—first stated in the Ebers Papyrus, dating to approximately 1550 BCE—that employed plant extracts, animal fats, and natural adhesives to fabricate simple patches for wound healing and pain relief [3], modern epidermal patches have progressed far beyond these physical barriers and stand as sophisticated systems for drug delivery, biosensing, and physiological monitoring [4, 5]. Their ability to adhere effortlessly to the skin's surface without inducing irritation or discomfort renders them ideal candidates for continuous, non‐invasive biomedical use [6].

Based on this evolutionary route, many kinds of epidermal patches are currently available, ranging from traditional protective coverings to sophisticated drug delivery systems and biosensing platforms – each designed for a particular biomedical purpose [7, 8, 9]. These patches differ in their intended uses, structural designs, materials, and mechanisms of action [10]. One of the first and simplest types is a conventional patch acting as a physical barrier by shielding wounds from external contaminants, promoting healing, and providing mechanical support to damaged skin [11]. Adhesive bandages and gauze pads are typical examples; they are simple and effective and are still used in both professional and homecare settings [12]. Although these formulations are devoid of any active medicinal ingredients, they are widely recognized for wound care management due to their ability to reduce the risk of infection and accelerate the body's natural healing process [13]. On the other hand, drug delivery patches are developed to facilitate the controlled release of therapeutics offering advantages over oral or injectable routes such as prolonged drug release, bypassing of first‐pass metabolism, and improved patient compliance [12].

Prominent examples encompass transdermal patches such as scopolamine for motion sickness, which was approved by the Food and Drug Administration (USA) in 1979, followed by nicotine patches for smoking cessation in 1991 and fentanyl patches for chronic pain management, all of which administer active chemicals directly into systemic circulation through advanced transdermal delivery technologies [14]. Following this technological progression, sensing and monitoring patches have emerged, integrating biosensors that collect real‐time physiological data such as glucose level, sweat composition, and heart rate [15]. These devices represent a significant advancement in wearable healthcare by enabling continuous, non‐invasive monitoring that supports early diagnosis and personalized treatment strategies. Recent reviews have emphasized this shift toward intelligent, sensor‐integrated wound management systems, where flexible biosensors and smart bandages enable continuous biochemical monitoring of wound environments [16, 17, 18, 19].

Furthermore, implantable patches extend the functionality of epidermal systems beneath the skin surface [20]. Frequently utilized in cardiovascular or orthopedic contexts [21], these patches offer structural reinforcement or targeted therapeutic delivery within the body, bridging the gap between external wearables and implantable medical devices [22]. Lastly, microfluidic patches are a new platform as epidermal patch candidates that can change small amounts of biological fluids using microchannel networks [23]. These systems enhance the capabilities of epidermal platforms in personalized treatment by enabling precise diagnostics, targeted delivery of drugs, and the controlled collection of sweat and interstitial fluid (ISF) [24]. The microchannels, typically fabricated as micrometer‐scale grooves or embedded capillary structures within flexible polymer substrates, allow for passive or active transport of body fluids [25]. Once applied to the skin, they can collect sweat or ISF through surface tension or micro‐pumping mechanisms [26], directing the fluid toward integrated sensing regions or drug reservoirs [27]. In diagnostic applications, analytes present in these fluids interact with on‐chip biosensors, enabling real‐time biochemical analysis [28, 29]. For therapeutic use, the same channels can facilitate spatially controlled drug delivery by distributing compounds across targeted skin areas [30, 31]. This integration of microfluidics into epidermal patches represents a crucial step toward compact, closed‐loop health monitoring systems [32].

To ensure user convenience and safety and to fulfill their intended biomedical functions, epidermal patches must meet several critical design criteria. Ideal patches are engineered to seamlessly align with the skin's unique biological and mechanical environment, requiring overall skin‐patch compatibility which includes flexibility, mechanical durability, biocompatibility, and breathable adhesion [24]. For instance, they should accommodate natural skin deformations of up to 30%–70% strain, particularly over joints and other mobile areas, while maintaining long‐term contact without mechanical or chemical degradation [33, 34]. This performance is typically achieved by using stable, biocompatible, and sometimes biodegradable materials, including natural polymers (e.g., chitosan, alginate, hyaluronic acid) or synthetic alternatives (e.g., silicone elastomers, polyurethane), offering tunable mechanical and degradation profiles [35, 36]. For example, bioadhesive polysaccharide‐based microneedle patches incorporating chitosan matrices have demonstrated improved interfacial stability and sustained therapeutic delivery for skin repair applications [37]. Secure but gentle skin adhesion and moisture regulation are essential to prevent irritation or maceration, especially during extended wear [38, 39]. Additionally, advanced applications—such as biosensing or drug delivery—require minimal immune response and effective biointegration [40]. To address these multifaceted requirements, recent developments have explored hybrid material systems [41], the incorporation of functional nanoparticles [9], and novel fabrication techniques [42] to enhance performance and overcome traditional limitations.

Technologies such as microneedles (MNs) [43], hydrogel matrices [44], and flexible electronics [45] have enabled precise interaction with the skin, facilitating real‐time monitoring and personalized therapeutic interventions [46]. Recent advances in MN engineering have further expanded their potential, including breathable multifunctional MN patches designed for efficient wound healing and controlled drug release [47]. Fabrication techniques have also undergone considerable development in conjunction with these functional improvements [48]. The production of highly customizable patches with intricate architectures is now possible through 3D printing [49], 4D printing [50], electrospinning [51], and microfabrication techniques [52], which thus boosts performance, patient comfort, and adaptability to a variety of clinical standards [53]. The rapid expansion of the commercial landscape has reflected this technological advancement. The demand for non‐invasive, continuous health monitoring solutions is rising, as evidenced by the widespread availability of transdermal patches for drug delivery, biosensing wearables, and flexible electronic devices [54]. From 2023 to 2030, the global transdermal drug delivery market is predicted to increase at a compound annual growth rate (CAGR) of 4.5%. In 2022, the market was valued at about 6.2 billion USD [55]. Likewise, the ubiquitous biosensors market is rapidly expanding, with a projected increase from 20.1 billion USD in 2022 to over 80 billion USD by 2030 [56].

Numerous commercial products illustrate this trend; for instance, NicoDerm CQ (nicotine patch) by GlaxoSmithKline [57] and Duragesic (fentanyl patch) by Janssen Pharmaceuticals [58] are widely used for drug delivery, while Freestyle Libre by Abbott enables continuous glucose monitoring through a flexible, skin‐adherent sensor [59]. Moreover, the iRhythm Zio Patch provides an example of the clinical application of epidermal biosensing technologies for cardiac rhythm monitoring [59]. According to market trends, the combination of biosensing, data communication, and tailored analytics is redefining patient care models at the convergence of wearable technology and digital health ecosystems.

Despite significant progress in epidermal patches, current literature often addresses specific functionalities or isolated aspects of material design and application, without consolidating the broader technological landscape. For instance, recent studies have introduced a range of advanced patch platforms including hydrogel‐based MN patches for transdermal insulin delivery [60], graphene‐integrated biosensing patches for continuous glucose and lactate monitoring [61], stretchable electronic skin systems capable of multi‐modal physiological sensing [62], and multifunctional nanozyme‐integrated hydrogel patches capable of regulating inflammatory microenvironments and promoting tissue repair [63]. However, many of these works focus narrowly on single use‐cases or individual material innovations, lacking an integrated understanding of how these technologies interrelate or evolve collectively toward multifunctional, intelligent platforms.

The present review is intended to fill this gap by covering various aspects of epidermal patches, including their fabrication strategies, material properties, current and prospective applications. The main types of epidermal patches are classified based on their material composition and functional modalities, encompassing hydrogel‐, polymer‐, and electronic‐based systems, as well as hybrid platforms that integrate sensing, therapeutic, and regenerative functions. The most prominent healthcare applications include continuous physiological monitoring, controlled drug delivery, wound management, and tissue repair, among others. While our overview aims to be comprehensive, selectivity was required in highlighting pivotal studies. By synthesizing advancements in fabrication techniques, clinical applications, and market developments, this work offers a comprehensive and timely reference for the field. Moreover, we highlight emerging concepts such as AI‐driven biosensing, self‐healing materials, and autonomous therapeutic systems which are shaping the next generation of smart skin interfaces. This integrated perspective will guide researchers, clinicians, and industry stakeholders in advancing epidermal patch technologies toward next‐generation, intelligent healthcare solutions.

## 2 General Requirements of an Epidermal Patch

The performance of epidermal patches largely depends on their mechanical robustness, user comfort, and long‐term stability. Critical attributes such as toughness, stretchability, self‐healing capacity, and adhesive strength enable these patches to adapt seamlessly to the body's curvature and to preserve both structural integrity and functional performance.

Regardless of the specific material platform, whether hydrogel‐based, microneedle array, or electronic skin, effective epidermal patches must satisfy a core set of requirements to function reliably on the skin. These include mechanical compliance to accommodate skin deformation, adequate adhesion for sustained attachment, biocompatibility to avoid adverse reactions, and, for active devices, appropriate permeability or conductivity depending on the intended application. While different material classes offer distinct advantages, hydrogels have emerged as a particularly versatile platform due to their unique combination of tunable mechanical properties, high water content, and inherent biocompatibility. Consequently, many of the design principles discussed below are illustrated through hydrogel‐based systems, though the underlying requirements apply broadly across epidermal patch technologies.

### 2.1 Flexibility and Stretchability

Human skin, with a collagen content exceeding 50%, exhibits anisotropic and viscoelastic behavior, characterized by a Young's modulus of 57–140 MPa and a fracture strain limit of 25%–70% [64]. These mechanical features enable the skin to undergo substantial deformation during body movements while maintaining its sensory, protective, and regulatory functions. To ensure stable integration with the body, epidermal patch substrates must therefore possess mechanical properties comparable to those of human skin [65, 66]. However, conventional polymer hydrogels are often limited by brittleness and low strength because of their permanently crosslinked and heterogeneous networks [67]. The irregular distribution of crosslinks and the nonuniformity of polymer chain lengths in these networks diminish their ability to dissipate mechanical energy, resulting in low strength and toughness. To address this, recent studies emphasize precise control over crosslinking density and chain‐length uniformity to achieve robust and highly stretchable hydrogel networks [68, 69, 70].

**FIGURE 1:**
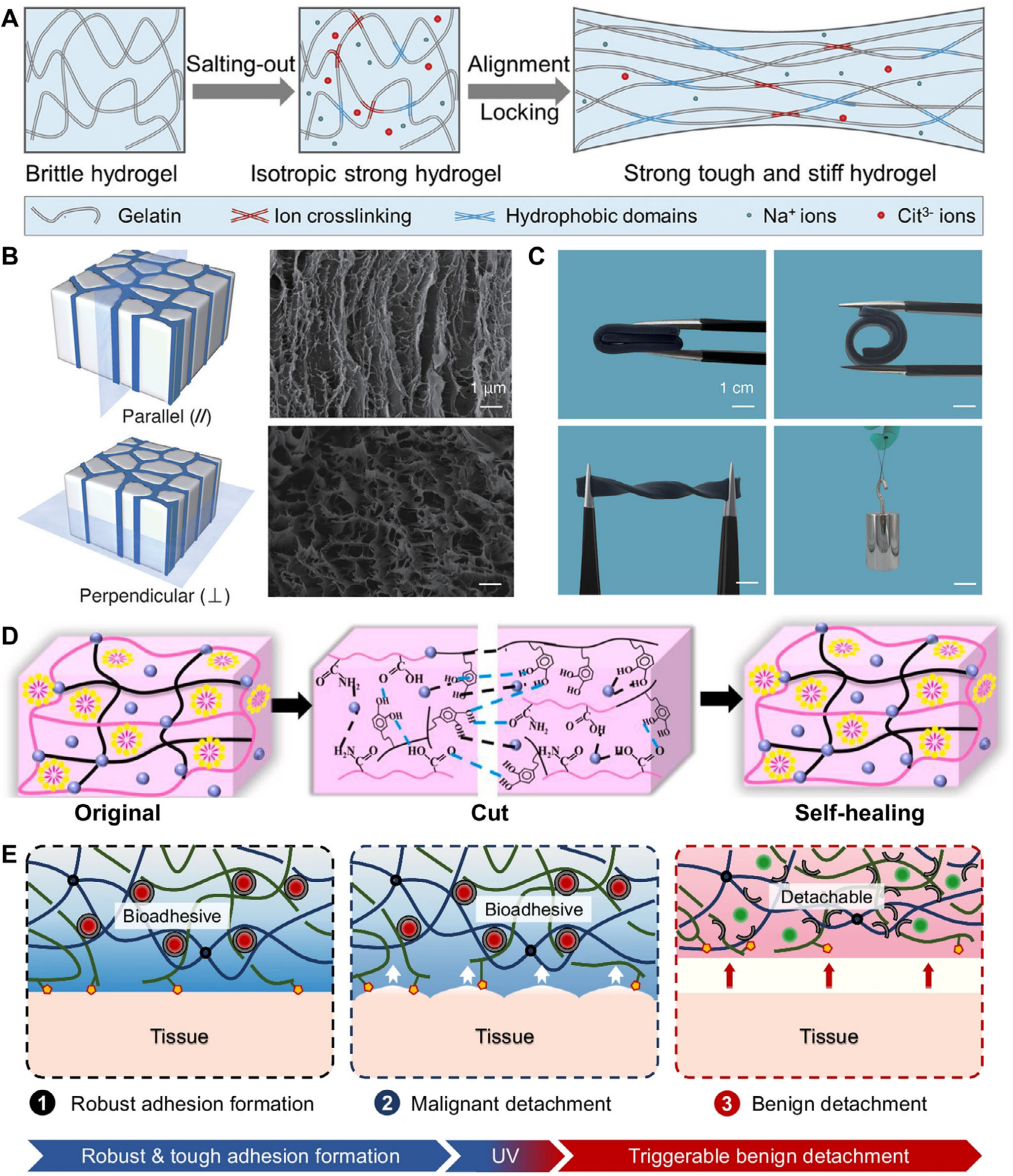
(A) Scheme of gelatin hydrogel during SALT processing. (Reproduced with permission [71]. Copyright 2024, Wiley‐VCH) (B) SEM images of the DFS PEDOT: PSS‐PVA hydrogels in parallel (∥) and perpendicular to (⊥) the direction of the ice template. (C) Images of DFS PEDOT: PSS‐PVA hydrogels subjected to folding, rolling, and twisting, displaying superior softness and flexibility. The hydrogels demonstrated high strength along the alignment direction, by sustaining a weight of 1 kg. (Reproduced with permission [72]. Copyright 2023, Wiley‐VCH) (D) Schematic diagram for the healing mechanism of PDA/SC/P(AM*‐co*‐AA)/Al^3+^ hydrogels. (Reproduced with permission [75]. Copyright 2024, Elsevier) (E) Schematic representation of a reversible configuration of the adhesive and photo‐detachable dynamic hydrogel with light‐activated supramolecular network. (Reproduced with permission [76]. Copyright 2024, Springer Nature).

Inspired by spider silk, Sun et al. introduced a “Salting‐Out—Alignment—Locking” (SALT) strategy to reinforce gelatin hydrogels (Figure 1A). By combining salt‐induced hydrophobic domain formation with prestretching‐induced chain alignment, the resulting anisotropic structure mimicked natural silk and conferred markedly enhanced tensile strength (10.12 MPa), Young's modulus (34.26 MPa), and toughness (14.28 MJ m^−3^), enabling directional flexibility for stretchable electronics [71]. In parallel, Zhang et al. reported fatigue‐resistant conducting polymer hydrogels using a combination of directional freeze‐casting and salting‐out techniques (Figure 1B,C). This approach created anisotropic microstructures within PEDOT: PSS/PVA hydrogels, yielding high stretchability (>600%), low Young's modulus (∼100 kPa), ultralow hysteresis, and rapid response times (130 ms) suitable for strain sensors in underwater robotics [72]. Although progress has been made, maintaining structural stability under repeated mechanical loading and achieving scalable, economical fabrication continue to pose difficulties.

On the other hand, Wu et al. demonstrated broad‐range tunable mechanical properties in PVA hydrogels through the Hofmeister effect, where specific ions induced polymer chain aggregation and network reinforcement (Figure 1D). This strategy produced highly tough (150 MJ m^−3^) and stretchable (2100%) hydrogels with Young's modulus values adjusted from 24 to 2500 kPa, covering the full range of soft biological tissues, while preserving high water content [73]. Building on this concept, Ren et al. engineered a non‐swelling zwitterionic hydrogel developed a non‐swelling zwitterionic hydrogel by promoting PVA crystallization and dense network formation via sulfobetaine methacrylate (SBMA) polymerization in acidic conditions [74]. The material combined high toughness with long‐term mechanical stability in seawater, along with ionic conductivity sufficient for underwater sensing. Nevertheless, the reliance on H_2_SO_4_ during synthesis raises concerns regarding safety and environmental impact. Exploring alternative routes that avoid strong acids, along with comprehensive biocompatibility assessments in marine environments, will be essential to ensure ecological safety and translational feasibility.

### 2.2 Self‐Healing Ability

Self‐healing is a critical property for epidermal patches, which are frequently subjected to mechanical stress during daily use, often resulting in micro‐damage that compromises performance. Epidermal patches with self‐healing properties can quickly repair cracks, scratches, or other damage through chemical reactions, physical changes, or other mechanisms, maintaining the integrity and performance of the epidermal patches [75]. Zhao et al. designed a mussel‐inspired hydrogel incorporating polydopamine (PDA)/sodium caseinate (SC) and poly(acrylamide‐*co*‐acrylic acid) (P(AM‐*co*‐AA)) crosslinked with Al^3+^ [77]. The self‐healing capability stemmed from dynamic hydrogen bonding, metal–ligand coordination, and π–π stacking, which collectively supported rapid recovery while preserving conductivity and mechanical robustness (Figure 1D). These reversible interactions enabled rapid recovery (97% healing efficiency within 1 h) while maintaining high conductivity (27 S m^−1^) and mechanical strength (1930 kJ m^−3^ toughness). In addition, Wang et al. introduced a ternary polyvinyl alcohol with strength elements (PVA‐S) hydrogel reinforced with hydroxypropyl cellulose (HPC) short‐chain fibers and carbon nanotubes (CNTs) [78]. The hierarchical network architecture, integrating dynamic hydrogen bonds with reinforcing nanofillers and crystalline domains, enabled rapid recovery from both surface scratches and bulk damage. This synergistic design conferred not only superior mechanical resilience but also intrinsic recyclability, thereby reinforcing its potential for next‐generation epidermal patches. Taken together, the contributions of Zhao et al. and Wang et al. underscore how bio‐inspired and hybrid hydrogel architectures can serve as a platform technology for next‐generation epidermal patches. By uniting self‐healing capability with mechanical robustness and electrical conductivity, these materials embody the essential characteristics of wearable bioelectronics. Looking ahead, systematic studies on their long‐term stability and skin integration will be crucial to translate this platform into diverse applications, from flexible electronics to advanced medical sensing systems.

### 2.3 Self‐Adhesiveness

Emerging self‐adhesive hydrogels are redefining the interface between electronics and biological tissues, moving beyond simple attachment to enable intelligent, conformal, and stimulus‐responsive integration [66, 70]. These materials function not merely as passive adhesives, but as active, dynamic interfaces capable of on‐demand attachment and detachment. It is a critical advancement for long‐term and user‐friendly wearable devices [79, 80, 81].

Zhang et al. developed a cellulose nanofiber (CNF)‐mediated supramolecular hydrogel that exhibits robust reversible adhesion and easy photodetachment [76]. As shown in Figure 1E, the hydrogel benefits from the reinforcement of CNF networks and the coordination between Fe^3^^+^ ions and polymer chains, which together enable dynamic reconfiguration of the supramolecular network and control of adhesion behavior. Specifically, the hydrogel demonstrates exceptional mechanical properties, including a maximum tensile stress of 0.053 MPa at a fracture strain of 1425%, and remarkable adhesion performance with interfacial toughness reaching up to 94.92 J m^−2^ on glass and 89.13 J m^−2^ on skin. Notably, this hydrogel allows for on‐demand detachment under UV light irradiation, with adhesion strength decreasing by over 92% after UV exposure. Its ability to maintain strong adhesion to various substrates while enabling gentle detachment with UV light makes it highly promising for applications in self‐powered electronic skins and wearable devices. In contrast, Hou et al. drew inspiration from the underwater adhesion mechanisms of mussels, incorporating catechol derivatives and hydrophobic alkyl monomers into hydrogel networks [82]. The synergistic effect of these components enabled the hydrogel to disrupt the hydration layer at the adhesive interface, allowing catechol groups to bind directly to substrate surfaces. Short alkyl chains were found to enhance wet adhesion by repelling water, while longer chains formed hydrophobic entanglements within the network, which limited mobility and reduced adhesion strength. The optimized hydrogel, containing short alkyl chains, demonstrated robust wet adhesion and was applied for advanced epidermal patches. Additionally, incorporating conductive nanocomponents allowed the hydrogel to work as a wearable device for continuous monitoring of physiological signals, such as electrocardiograms (ECG), even during swimming or on dynamic organs like beating hearts.

The self‐adhesive hydrogels developed by Zhang et al. and Hou et al. exemplify how drawing inspiration from natural adhesion mechanisms can lead to innovative materials with broad applications. Both studies highlight the importance of understanding and mimicking biological strategies, such as the dynamic non‐covalent interactions and mussel‐inspired catechol chemistry, to create materials that adhere strongly even in challenging conditions. The versatility of these hydrogels, combined with their ability to maintain adhesion under deformation and in wet environments, positions them as ideal candidates for next‐generation epidermal patches. Future work could focus on enhancing the conductivity and sensitivity of these materials for more advanced biosensing applications, further bridging the gap between wearable technology and human physiology.

### 2.4 Conductivity

The essential characteristic of epidermal patches is their ability to sense and respond to the external environment, and the conductivity of the material is a key factor in achieving this goal [86, 87]. Two general strategies can be adopted in this regard. The first involves integrating inherently conductive and stretchable conductors. The second entails physical processing or chemical techniques to incorporate conductive fillers, mainly including spin coating, 3D printing, electrodeposition, and in situ polymerization [88].

#### 2.4.1 Integrating Inherently Conductive Materials

**FIGURE 2:**
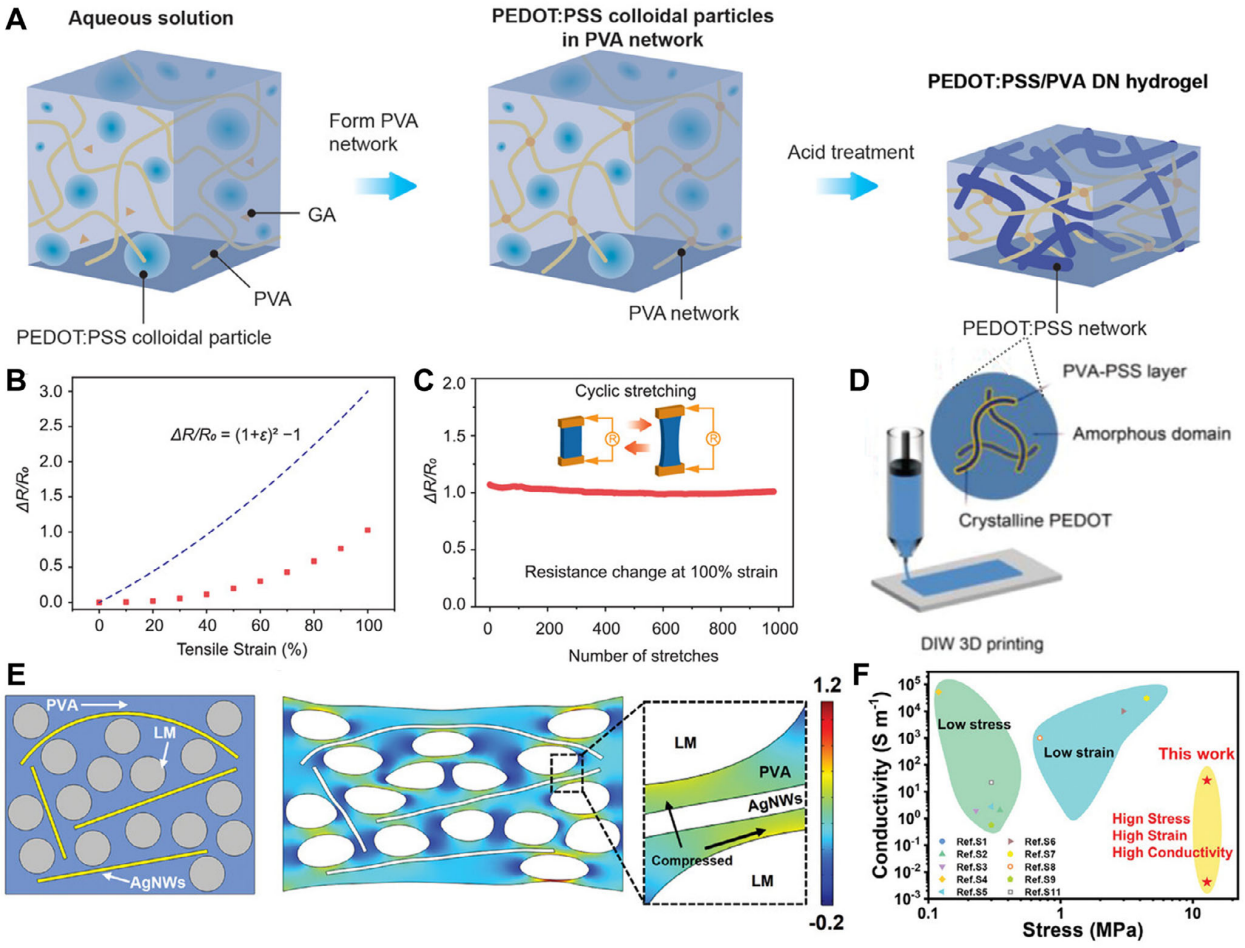
(A) Schematic representation of the fabrication process and the structural alterations of PEDOT: PSS and PVA during the crosslinking and the acid treatment. (B) Normalized change in resistance as a function of strain of the PEDOT: PSS/PVA DN hydrogel, and that for an ideal incompressible elastic conductor is present as a reference. (C) Normalized change in resistance of the PEDOT: PSS/PVA DN hydrogel loaded to 100% strain over 1000 cycles. (Reproduced with permission [83]. Copyright 2022, Wiley‐VCH) (D) 3D‐printing process of the PEDOT: PSS‐PVA conducting polymer hydrogel. (Reproduced with permission [84]. Copyright 2022, Wiley‐VCH) (E) 2D finite element analysis models of the PAL hydrogel at 0% and 40% strain after being calculated. The color legend represents the volumetric strain. The PVA matrix between the LM and AgNWs was obviously compressed, resulting in a decrease in the distance between the LM and AgNWs. (F) Comparison of mechanical properties and conductivity between PAL and reported high‐conductivity hydrogels. (Reproduced with permission [85]. Copyright 2024, Wiley‐VCH).

Inherently conductive polymers (ICPs) such as PEDOT: PSS have been widely employed to improve the electrical performance of hydrogels. In a study, Li et al. developed a highly conductive and stretchable double‐network hydrogel by integrating high‐content PEDOT: PSS with PVA through in situ polymerization and densification (Figure 2A) [83]. The acid treatment promoted PVA chain compaction and PEDOT: PSS aggregation, yielding a continuous conductive network with excellent stretchability and stable electrical response under cyclic strain. Conductivity and mechanical performance were further enhanced by an acid‐induced densification step, in which acetic acid promoted PVA chain compaction and PEDOT: PSS aggregation, leading to a continuous conductive network within the PVA matrix. As a result, the hydrogel displayed stable electrical conductivity under strain, with normalized resistance changes (Δ*R*/*R*_0_) of only ∼0.2 at 50% strain and 1.1 at 100% strain (Figure 2B), while maintaining high stretchability and cycling stability over 1000 stretch–release cycles (Figure 2C). Similarly, Chong et al. have reported the template‐directed assembly of PEDOT: PSS fibers within a chemically crosslinked soft polymeric network, producing the so‐called T‐CH (electrically conductive hydrogel through template‐directed assembly) hydrogel [89]. This material exhibited tissue‐like Young's modulus (∼25 kPa), high stretchability (610%), toughness (1 MJ m^−3^), high water content (90 wt.%), and record‐high conductivity (247 S cm^−1^), making it a promising conductive tissue‐interfacing platform. In another approach, Shen et al. fabricated PEDOT: PSS‐PVA hydrogel strain sensors using 3D printing, where printable conductive polymer ink was prepared by mixing PEDOT: PSS nanofibers with PVA solution (Figure 2D) [84]. The 3D printing process allowed precise structural control, enabling rapid fabrication of complex geometries and optimization of both mechanical and electrical performance. The 3D‐printed hydrogel strain sensors exhibited remarkable stretchability (∼300%), ultralow hysteresis (<1.5%), and improved conductivity, underscoring their promise for next‐generation wearable and flexible electronics.

Beyond PEDOT: PSS, polyaniline (PANI) and polypyrrole (PPy) have recently emerged as complementary ICPs that can further broaden the property space of conductive hydrogels. Devi et al., for example, combined repetitive freeze–thaw cycles with in situ PANI polymerization to generate a PANI/PVA double‐network film that displays a skin‐matched modulus (35 kPa), an interconnected pore hierarchy (78 m^2^ g^−1^ surface area) and a room‐temperature conductivity of 28 S m^−1^ [90]. On the PPy side, a higher electrical performance was demonstrated by He et al., who used an aramid‐nanofiber (ANF) percolation template to direct PPy polymerization into a hyper‐connected, nanofibrous network [91]. The resulting ANF‐PPy hydrogel delivers an exceptional conductivity of 72 S cm^−^^1^, a fracture strength of 27.2 MPa, and a specific capacitance of 240 F g^−^^1^ while maintaining <2% resistance change after 10 000 charge–discharge cycles or bending to a 0.5 mm radius. Patterned ANF‐PPy bioelectrodes exhibit sub‐20 Ω cm^2^ impedance at 1 kHz and have been used to record high‐fidelity ECG signals and to pace cardiomyocytes at 2 Hz, illustrating the potential of PPy‐based systems for both energy‐storage and electrophysiological interfaces.

These examples highlight the value of coupling conductive polymers with soft matrices and leveraging advanced fabrication strategies to meet diverse application demands. The synergy of conductivity, stretchability, and durability achieved in these systems points to their strong potential for wearable electronics, flexible sensors, and bioelectronic interfaces. Looking ahead, future work should prioritize scalable processing, hybridization with multiple conductive systems, and improvements in biocompatibility and environmental stability to accelerate translation into real‐world applications.

#### 2.4.2 Incorporating Conductive Fillers

**FIGURE 3:**
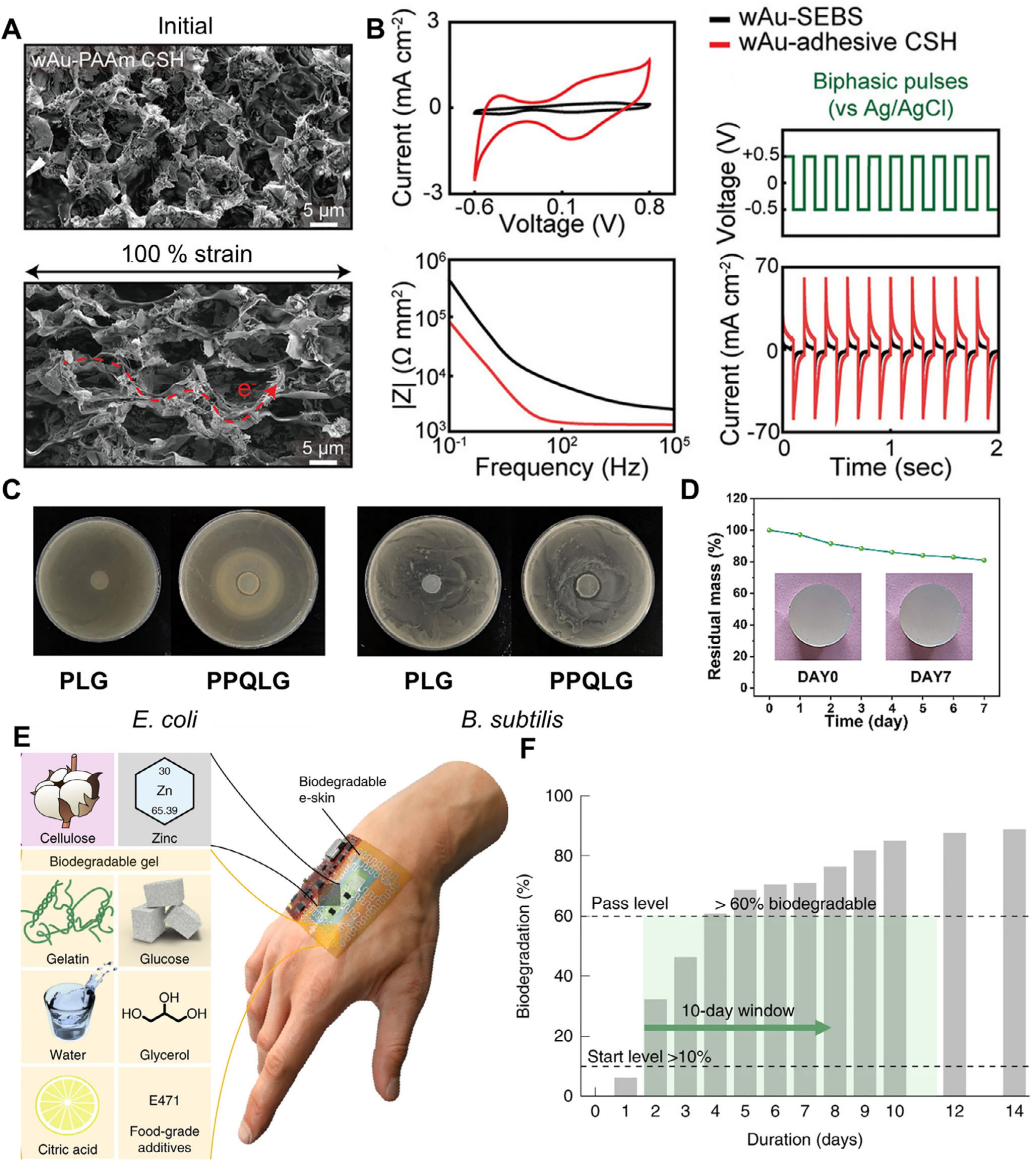
(A) SEM images of wAu‐polyacrylamide (wAu‐PAAm) conductive and stretchable hydrogel (CSH) before and after stretching. (B) Electrochemical properties of wAu‐SEBS (black) and wAu‐adhesive CSH (red). Cyclic voltammetry, electrochemical impedance spectroscopy, and chronoamperometry results in PBS solution. A biphasic voltage pulse (green) is applied to the electrode in chronoamperometry. (Reproduced with permission [93]. Copyright 2024, Wiley‐VCH) (C) Antibacterial zone demonstration of the PPQLG hydrogel against *Escherichia coli* and *Bacillus subtilis*. (D) Pictures and tests of water retention properties of the PPQLG hydrogel. (Reproduced with permission [94]. Copyright 2024, Elsevier) (E) Naturally derived ingredients, such as gelatin and citric acid, enable an elastic and stable, but fully degradable, biogel. (F) Complete aerobic decomposition of the biogel, measured via biological oxygen demand (BOD) of the microorganisms in the test solution (wastewater). The pass level for ready biodegradability is the removal of 60% of dissolved organic compounds in a 10‐day window. (Reproduced with permission [95]. Copyright 2020, Springer Nature).

In addition to conductive polymers, the integration of conductive fillers such as liquid metal particles, conductive polymers, MXene, carbon nanotubes, and graphene into the hydrogel matrix offers another effective technique to enhance the electrical performance of hydrogels [92]. Wang et al. reported a tough, conductive hydrogel by integrating silver nanowires (AgNWs), liquid metal (LM), and PVA [85]. LM particles were ultrasonically dispersed in ethanol, mixed with AgNWs in a PVA solution, and concentrated by water evaporation to increase chain density and entanglement. Under strain, Poisson's effect induced matrix contraction, aligning LM particles and AgNWs into conductive pathways (Figure 2E), which boosted conductivity from 4.05 × 10^−^^3^ to 24 S m^−1^ a ∼6000‐fold enhancement. Thus, PAL hydrogels resolve the long‐standing trade‐off between toughness—that is, simultaneously elevated strain and stress—and conductivity, achieving better mechanical and electrical performances than those of other conductive hydrogels (Figure 2F). In another study, Lim et al. engineered a highly conductive and stretchable hydrogel (CSH) nanocomposite by incorporating whiskered gold nanosheets (W‐AuNSs) into various hydrogel matrices (Figure 3A) [93]. The integrated AuNS framework enabled efficient electron‐ion transduction even under high water content, yielding superior electrochemical conductivity compared with poly(styrene‐ethylene‐butylene‐styrene) (SEBS) elastomer‐based nanocomposite (wAu‐SEBS) (Figure 3B). As a result, the gold‐conductive hydrogel demonstrated superior performance in epidermal patches compared to conventional elastomer‐based materials.

Looking ahead, the exploration of novel conductive fillers and hybrid systems holds considerable potential for advancing hydrogel performance. Synergistic combinations of filler types could further boost conductivity and mechanical robustness, while progress in nanotechnology may enable precise control over filler distribution and interfacial interactions. Beyond current applications in wearable devices, these strategies could extend to energy storage, bioelectronic interfaces, and advanced sensor technologies. As the field evolves, the integration of conductive fillers will remain central to shaping next‐generation flexible and wearable electronics.

#### 2.4.3 Performance Trade‐Offs and Application Matching of Conductive Material Systems

The two mainstream conductivity regulation strategies outlined above, ICP integration and conductive filler incorporation, exhibit distinct performance profiles and inherent trade‐offs across the core metrics required for epidermal patches, namely conductivity, stretchability, long‐term stability, biocompatibility, and processability. For ICP systems, PEDOT: PSS delivers the most balanced overall performance, with wide processing compatibility for additive manufacturing, stable electrical response under cyclic strain, and well‐validated biocompatibility for bioelectronic interfaces. Its key trade‐off lies in the inherent conflict between high conductivity and stretchability, as high‐content PEDOT tends to aggregate and reduce network flexibility, alongside relatively high raw material costs [83, 84, 89]. In comparison, PANI offers a low‐cost alternative with excellent environmental robustness, but its conductivity is highly pH‐dependent, with irreversible performance degradation under the neutral/weakly alkaline conditions of skin physiological environments. Moreover, its intrinsic brittleness requires extensive composite modification to meet the stretch requirements of epidermal wear [90]. PPy stands out for its superior biocompatibility, long‐term cycling stability, and ultra‐low interfacial impedance for electrophysiological monitoring, yet its poor processability and intrinsic rigidity severely limit scalable fabrication of large‐area or structurally complex patches [91].

For conductive filler composite systems, the core advantage lies in the flexible tunability of performance via filler type and loading, yet each system faces unique performance trade‐offs. Liquid metal‐based composites uniquely break the traditional “strain‐induced conductivity decay” dilemma, with conductivity increasing significantly under deformation to suit high‐strain scenarios such as joint‐mounted patches, but suffer from high material costs, poor compatibility with hydrogel matrices, and long‐term leakage risks [85]. MXene‐based composites offer ultra‐high intrinsic conductivity and integrated multifunctionality such as photothermal antibacterial effects, but their poor oxidation resistance in aqueous physiological environments leads to rapid conductivity loss, making them unsuitable for long‐term continuous wear. Carbon‐based fillers (carbon nanotubes, graphene) provide excellent chemical stability and mechanical reinforcement with low cost and scalable production potential, but are prone to agglomeration in hydrogel networks, with high loadings markedly reducing stretchability [92]. Noble metal fillers (silver nanowires, gold nanosheets) enable ultra‐high‐sensitivity electrochemical biosensing, with gold‐based materials offering gold‐standard biocompatibility, but their prohibitive cost limits their widespread use in general‐purpose patches. Collectively, these performance trade‐offs define the application boundaries of each conductive system, providing a clear selection framework for the design of epidermal patches to specific application scenarios in subsequent sections [93].

### 2.5 Biocompatibility and Safety Issues

Biocompatibility is a prerequisite for epidermal patches, ensuring reliable performance under diverse biochemical conditions and minimizing adverse immune responses [94]. However, the moist microenvironment of epidermal patches often favors bacterial growth, elevating the risk of infection and inflammation at damaged sites [95]. Therefore, integrating antimicrobial functionality is a central requirement in designing safe and durable wearable systems. Zhang et al. developed a multifunctional conductive hydrogel (PPQLG) incorporating quaternary ammonium chitosan salt (QCS) and polyhexamethylene biguanide hydrochloride (PHMB) [94]. QCS disrupts bacterial cell wall integrity by altering the pH, while PHMB enhances antibacterial activity by increasing membrane permeability. The hydrogel displayed strong antibacterial effects against *E. coli* and *B. subtilis*, producing inhibition zones of 2.1 and 3.3 mm, respectively (Figure 3C), and showed excellent biocompatibility with no adverse tissue response after implantation in mice for 14 days. Additionally, the incorporation of glycerin and lithium chloride further endowed the system with antifreeze and water‐retention capacity (Figure 3D), maintaining >80% remaining mass after 7 days under ambient conditions. In a complementary approach, Baumgartner et al. reported a fully degradable gelatin‐based biogel that combines resilience with epidermal patches and soft robotics [95]. Fabricated from gelatin, citric acid, and cellulose (Figure 3E), this biogel retained its mechanical properties for over a year and tolerated >330 000 folding cycles without failure. It was also self‐adhesive, rapidly self‐healing, and enzymatically degradable by wastewater bacteria within five days (Figure 3F). Importantly, its food‐grade constituents ensured biocompatibility and eco‐friendliness, offering a sustainable platform for epidermal patch applications. Taken together, these advances emphasize that future material design must balance biocompatibility, antimicrobial protection, and environmental sustainability. Such multifunctional systems hold the potential to define the next generation of safe, durable, and eco‐conscious epidermal patches for medical and wearable technologies.

## 3 Fabrication of Hydrogel Patches

**FIGURE 4:**
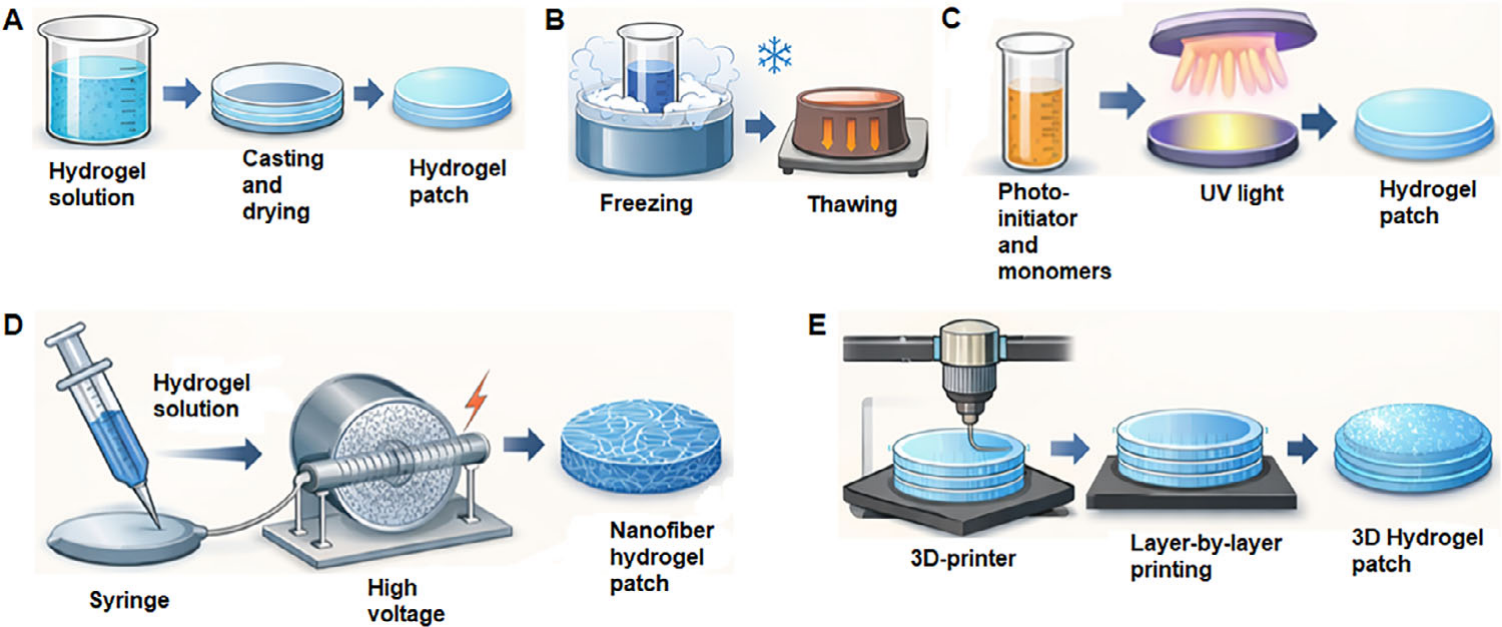
Schematic illustrating different techniques for the fabrication of hydrogel patches: (A) solvent casting and evaporation; (B) freeze–thawing; (C) photo‐polymerization; (D) electrospinning; (E) 3D printing technology. Created with Biorender.com.

Hydrogel patches are fabricated via multiple techniques, with 3D printing and photo‐polymerization being the most advanced (Figure 4). The choice depends on drug compatibility, mechanical needs, and scalability. Future advancements may focus on smart hydrogels (stimuli‐responsive) and AI‐optimized fabrication [96].

The solvent casting and evaporation technique is among the simplest and widely used approaches for fabricating hydrogel patches. In this technique, the polymer is dissolved in a suitable solvent; thereafter, the solution is cast into a mold, allowing the solvent to evaporate, leaving behind a solid hydrogel film [97]. This technique is principally useful for drug‐loaded hydrogel patches due to its simplicity, scalability, and compatibility with various polymers and active compounds. If evaporation is uneven, this technique is often constrained by poor mechanical strength, potential solvent residue, slow drying time, and non‐uniform thickness. Numerous studies have explored the solvent casting and evaporation techniques using natural and synthetic polymers for the preparation of hydrogel patches [98]. Although this technique has been widely utilized in the pharmaceutical and polymer sectors that employs industrial‐scale casting machines and drying tunnels. It is simple and cost‐effective, relying on minimal, affordable equipment. Uniform thickness is easily achieved by controlling the cast solution's volume. The process yields transparent patches with good optical clarity, aiding wound inspection and ensuring batch consistency. However, drawbacks include potential toxic solvent residues, requiring strict, slow drying. It is limited to thin films and can produce brittle patches without plasticizers. The lengthy evaporation phase and potential degradation of heat‐sensitive drugs due to poor encapsulation efficiency are additional limitations.

The freeze–thawing is another technique based on a physical crosslinking technique without chemical crosslinkers. This technique relies on repeated freezing and thawing cycles to induce crystallinity and polymer chain entanglement, forming a stable 3D network. In this approach, a hydrophilic polymer (e.g., PVA or PVP) is dissolved in water, and the solution is frozen (typically at −20°C to −80°C) to form ice crystals, pushing the polymer chains into concentrated regions. Thereafter, the frozen solution is thawed (at room temperature or 4°C) to allow the polymer chains to reorganize, forming physical crosslinks via hydrogen bonds and crystalline domains. To improve the mechanical strength of the hydrogel materials, it is subjected to multiple freeze–thaw cycles (3–10 cycles). This technique offers medium to high scalability but relies on specialized freeze–thaw equipment, creating a potential drawback. It is an environmentally friendly, solvent‐free process using water, eliminating toxic chemical crosslinkers. The resulting patches boast high mechanical strength, elasticity, and a porous structure ideal for cell infiltration and rapid fluid absorption. Moreover, the low processing temperatures preserve the integrity and bioactivity of sensitive therapeutic agents. While prolonged processing time may take several hours or days, limited to specific polymers (like PVA), and low porosity without the incorporation of additives serves as hindrance to this technique; the high‐water retention and flexibility, tunable mechanical strength, biocompatibility due to the lack of chemical crosslinkers, and suitability for sensitive drugs are the major highlights that promotes the wide acceptance of this technique in many biomedical applications (Table 1). However, the microcrystalline‐induced opacity may constrain cosmetic applications, and the technique is largely limited to polymers, such as PVA, that can form stable crystalline domains.

**TABLE 1:** Summary of studies that explored the freeze–thawing technique for fabricating hydrogel patches.

Materials	Freeze conditions	Thawing conditions	Application	Key findings	Ref.
PVA/chitosan/PVP	Three cycles of freezing at −20°C for 10 h	Room temperature (RT), 4 h	Transdermal delivery of high‐dose drugs	Improved drug‐loading capacity, Remarkable hypoglycemic effect without causing any risk in diabetic rats up to 14 h of usage.	[99]
Natural herbal extracts, PVA, and propylene glycol	−76°C for 4 min	RT	Atopic dermatitis	Significantly enhanced healing due to long‐term moisturizing, non‐toxic and harmless to patients, can be easily attachable or detachable from the skin, without any trace.	[100]
PVA/Dextran	−20°C for 16 h	20°C for 5 h	Delivery of antioxidants astaxanthin	*Ex vivo* and in vivo studies demonstrated favorable mechanical and compatibility characteristics, indicating potential as myocardial patches to assist infarcted heart mechanical function and reduce oxidative stress.	[101]
PVA/hFDM membrane (human lung fibroblast‐derived matrix)	−20°C for 24 h	RT, 1 h	Wound healing	The loading of ciprofloxacin into the hydrogel system suppressed the colony growth of bacteria, stimulated faster wound closure, and skin regeneration.	[102]
Carbamazepine and PVA	Five cycles of freezing at −20°C for 18 h	RT, 6 h	Transdermal delivery	An extended drug release profile of 85.2% and 59.6% permeation percentage over 96 h	[103]
PVA/chitosan/moringa‐extract	Five cycles of freezing at −40°C overnight	34°C for 30 min	Wound‐healing	The patch facilitates prompt release of components, tissue regeneration, and rapid thrombus formation; its anti‐inflammatory and antibacterial properties reduce healing time.	[104]
PVA and PEG	−20°C for a variable period	50°C	Drug loaded transdermal patches	Significant release of diltiazem hydrochloride compared with the thermally treated PVA membrane.	[105]
PVA, Poly(allylamine hydrochloride) (PAH), and 3‐carboxy‐4‐fluorophenylboronicacid (FPBA)	Three cycles of freezing at −20°C for 2 h	10°C for 1 h	Insulin delivery	Good glucose‐responsive insulin‐release ability.	[106]
PVA and chitosan	Three cycles of freezing at −20°C for 10 h	RT, 4 h	Skin ISF‐based point‐of‐care testing	Successful detections of standard indicators obtained from the mimetic skin and monitoring the glucose level in rabbit skin (>24 h).	[107]
Silver nanoparticles loaded PVA	Three cycles of freezing at −20°C for 6 days	20°C	Wound healing	Significant wound healing efficacy demonstrated through histopathological analysis of wound lesions at regular intervals.	[108]
PVA/silver nanocomposite	Three cycles of freezing at −20°C ± 2°C for 16 h	20°C ± 2°C, 2–6 h	Antimicrobial dressing scaffold	Effective antimicrobial retention after 96 h of release of AgNPs, thus, functioning as a reservoir for AgNPs to maintain a moist and sterile environment for a long time.	[109]
NaOH‐enhanced PVA	−20°C for 12 h	RT, 4 h	Penis enlargement	Can effectively enlarge the penis without degradation or fibrosis while maintaining long‐term stability in vivo.	[110]

Photo‐polymerization is another versatile technique for fabricating hydrogel patches using ultraviolet (UV) or visible light to initiate crosslinking. This technique employs a photocurable polymer (e.g., polyethylene glycol diacrylate (PEGDA), gelatin methacrylate (GelMA), or hyaluronic acid methacrylate (HAMA) mixed with a photoinitiator (such as Eosin Y) under UV‐light (365 nm) or visible light (405 nm, blue light) for about 5–60 s. The photoinitiator absorbs light, thereby generating free radicals that initiate polymerization. These free radicals could induce covalent bonding between the polymer chains to produce 3D hydrogel networks. This approach offers numerous advantages like fast curing rates (in seconds to minutes), high spatial resolution (useful for MNs, micro‐patterning), tunable mechanical and chemical properties of the hydrogel patch, and cell‐friendly biomaterials (if using cytocompatible photoinitiators) [111]. This medium‐scale technique often suffers from the use of photoinitiators, which pose toxicity risks; oxygen can cause tacky surfaces due to incomplete curing, and UV light struggles to penetrate thick materials. Specialized equipment and safety measures also increase costs.

Electrospinning is another technique for generating nanofibrous hydrogels with high surface area, porosity, tunable mechanical properties as well as functional properties for many biomedical applications [112, 113]. This unique technology uses electrostatic forces to draw polymer solutions into ultrafine fibers. Typically, the hydrogel precursor (e.g., PVA, alginate, gelatin) is dissolved in a suitable solvent, and loaded with functional additives (like drugs, nanoparticles, growth factors) for electrospinning under a high‐voltage power supply (5–30 kV). This scalable technique utilizes multi‐nozzle or needleless systems, though maintaining fiber uniformity during production remains challenging. It creates nanofibrous mats with a high surface area that mimic the extracellular matrix, promoting cell adhesion. The highly porous structure ensures breathability, enabling gas exchange while blocking fluids. This technique offers excellent controlled release for drug delivery, and fiber alignment can guide cell growth for tissue regeneration. A syringe pump is used to feed the polymer solution at a controlled rate (0.1–2 mL h^−1^), where a collector plate (stationary or rotating) gathers the generated nanofibers [114]. The nanofibers are formed like a Taylor cone when the electric field is injected into the system, overcoming surface tension. Finally, as the solvent evaporates, the dry hydrogel‐like fibers are deposited. This technique has been widely utilized for the fabrication of functional hydrogel patches for application in wound healing [115], peritendinous anti‐adhesion [116], and rheumatoid arthritis [117] (Table 2). Although this technique is highly advantageous, limitations such as solvent toxicity (e.g., DMF, chloroform), low throughput from single‐needle setups, specific polymer requirements, mechanically fragile mats that may require backing layers, and the need for post‐crosslinking for hydrogel stability, and batch‐to‐batch variability for humidity/temperature sensitive materials need to be overcome to obtain ultrafine nanofibers [118]. Therefore, it is imperative to explore a novel and green electrospinning approach using water‐based solvents, utilizing multi‐material coaxial electrospinning, near‐field, and in situ electrospinning to overcome these limitations [119, 120, 121].

**TABLE 2:** Recent advances in the synthesis and application of hydrogel patches based on the electrospinning technique.

Materials	Electrospinning parameters				Application	Key findings	Ref.
	Flow rate (unit)	Voltage (kV)	Collector distance (cm)	Speed (rpm)			
PCL@HA‐ADH@PA/Fe	0.2 mm min^−1^	20	15	2000	Prevention of peritendinous adhesion	Self‐healing, tissue adhesion, antibacterial activity, modulation of macrophage polarization, oxidative stress elimination, and anti‐inflammation.	[121]
PVA/β‑CD/AuNPs	0.6 mL h^−1^	15	20	NA	Wearable biosensor	Superior permeability to bioactive substrates, fast electron transfer, and improved sensing performance to low concentrations of glucose (0.01 mm) in human serum.	[122]
MeGel/PLLA	0.8 mL h^−1^	15	18	1800	Diabetic wound healing	Efficient hemostatic efficacy, faster wound healing (e.g., diabetic wounds), and regeneration.	[123]
PVP‐SiO_2_	0.1 mm min^−1^	15	20	∼3.2	Drug release	Outstanding moisture retention and gas permeability allow the skin to breathe while preventing water infiltration, complemented by strong adhesion for long‐term wear.	[124]
PANI/PLGA, PANI/PLGA/MWCNT	4 mm h^−1^	15	15	1200	Biomedical patch	High electrical conductivity, good thermal stability, and remarkable signal transduction for potential myocardial infarction repair	[125]
IPC extract‐loaded PVA	0.5 mL h^−1^	21	15	600	Infected wound	Enhanced antimicrobial activity against *S. aureus*, compared with a commercial dressing patch.	[126]
PHBV	0.8 mL h^−1^	15	18	NA	Diabetic wound healing	Sustained drug release behavior for nearly one month, promoted the proliferation of human dermal fibroblasts.	[127]
CAH/PLANF	3 mL h^−1^	20	18	NA	Chronic wound treatment	Photodynamic antibacterial properties, antimicrobial property and enhanced wound healing.	[128]
GelMA, CIP, PCL‐COL	1 mL h^−1^	18	12.5	NA	Tissue engineering	Degradation characteristics, controlled drug release, antibacterial efficacy, and cytocompatibility.	[129]
PVA, PLGA, CuS NPs, valsartan	1 mL h^−1^	15	20	NA	Skin wound healing	Good antibacterial, anti‐inflammatory, and sustained‐release properties.	[130]

PCL – poly(ɛ‐caprolactone), HA‐ADH – hyaluronic acid‐adipic acid dihydrazide, PA – protocatechuic aldehyde, PVA/β‑CD – poly(vinyl alcohol)/β‐cyclodextrin, MeGel – methacrylated gelatin, PLLA – poly (L‐lactic acid), PVP‐SiO_2_ – polyvinylpyrrolidone‐silica, PANI – polyaniline, PLGA – poly(lactic*‐co‐*glycolic acid), MWCNT – multi‐walled carbon nanotubes, IPC – *ipomoea pes‐caprae*, PHBV – poly(3‐hydroxybutyrate*‐co‐*3‐hydroxyvalerate), CAH/PLANF – calcium alginate hydrogel/polylactic acid nanofiber, GelMA – gelatin methacryloyl, CIP – ciprofloxacin, PCL‐COL – polycaprolactone‐collagen, CuS NPs – copper sulfide nanoparticles.

### 3.1 3D Printing Technique

**FIGURE 5:**
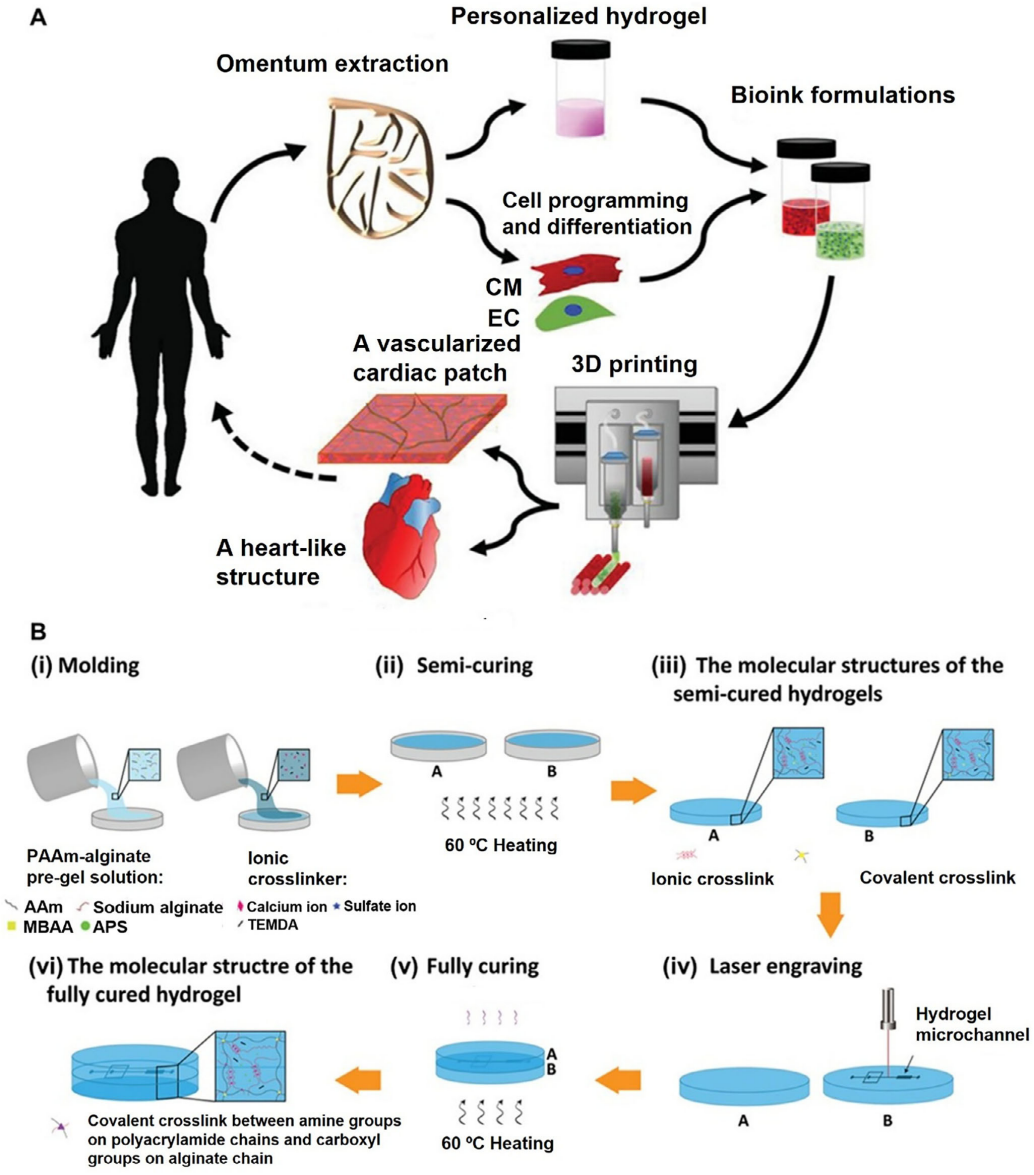
(A) Conceptual schematic illustrating the generation of personalized bioinks and the fabrication of vascularized cardiac tissues. Omentum‐derived cells are reprogrammed and differentiated into cardiomyocytes (CMs) and endothelial cells (ECs), combined with personalized hydrogels to form bioink formulations, which are subsequently used in 3D printing to produce vascularized cardiac patches and heart‐like structures. Reproduced with permission from ref. [162]. Copyright 2019, Wiley‐VCH. (B) Fabrication process of tough hydrogel‐based microfluidic devices. The process involves molding a polyacrylamide–alginate pre‐gel solution (AAm, MBA, APS, sodium alginate) followed by semi‐curing under heating (60°C) with ionic crosslinking. Laser engraving is then applied to create microchannels, after which full curing forms covalent crosslinks between amine and carboxyl groups, resulting in a robust hydrogel network. Reproduced with permission from ref. [163], Copyright 2019, Wiley‐VCH.

3D printing is an advanced fabrication technique for producing hydrogel patches with precise geometries, tunable mechanical properties, and embedded biological functionalities. The integration of 3D printing with hydrogel fabrication allows for the creation of patient‐specific patches for tailored biomedical applications (Figure 5A). One of the key advantages of 3D printing is its ability to control the spatial distribution of materials, which permits the fabrication of complex structures with high resolution. This technique involves the layer‐by‐layer deposition of hydrogel precursors, which are then crosslinked to form stable, three‐dimensional networks [131]. The process begins with the preparation of a hydrogel bioink, which must exhibit suitable rheological properties to ensure smooth extrusion and shape fidelity post‐printing. These bioinks are often formulated from natural polymers, including alginate, gelatin, hyaluronic acid, or fibrin, as well as synthetic polymers like poly(ethylene glycol) diacrylate (PEG‐DA), each providing different advantages regarding mechanical strength, biodegradability, and cellular interaction [132]. This technique is currently the least scalable for mass production, limiting it to prototyping and personalized medicine. Its key strengths include creating complex geometries like patient‐specific shapes, multi‐material printing, and high micron‐scale precision for on‐demand customization. However, it is also associated with some significant drawbacks. The layer‐by‐layer process is very slow, making high‐volume manufacturing uneconomical. High equipment and bio‐ink costs, stringent rheological material requirements, and the risk of damaging living cells during printing further hinder adoption. Post‐processing steps add additional complexity.

In this technique, once the bioink is prepared, it is loaded into a printing system where it is deposited in a predefined pattern based on a digital model. The printing process must carefully balance parameters such as extrusion pressure, nozzle diameter, and printing speed to achieve optimal resolution and structural integrity [133, 134]. After deposition, the hydrogel is stabilized through crosslinking, which can be achieved via various mechanisms, including ionic interactions [135], ultraviolet (UV) light exposure [136, 137], thermal gelation [138], or enzymatic reactions [139]. The choice of crosslinking method influences the hydrogel's mechanical properties, degradation rate, and biocompatibility, making it a critical consideration in patch design. A significant benefit of 3D‐printed hydrogel patches is their ability to incorporate bioactive molecules, such as growth factors, antibiotics, or anti‐inflammatory drugs, directly into the polymer matrix [140]. This enables controlled and sustained release, enhancing therapeutic efficacy in applications like chronic wound healing or localized drug delivery. Additionally, living cells can be embedded within the hydrogel during printing, creating cell‐laden constructs for tissue regeneration. For example, 3D cellular alignment can be performed by embedding a cell/collagen hydrogel into predefined electrohydrodynamically printed microlattices [141]. The precision of 3D printing ensures uniform cell distribution, promoting better tissue integration and functionality compared to traditional scaffold fabrication techniques.

Another notable application of 3D‐printed hydrogel patches is in the development of wearable biosensors. Conductive hydrogels, often enhanced with materials like graphene oxide, carbon nanotubes, or silver nanowires, can be printed into flexible, stretchable patches capable of monitoring physiological signals such as glucose levels, pH, or cardiac activity [142]. These patches offer a seamless interface with the skin, providing real‐time health monitoring without discomfort. Furthermore, the ability to customize patch geometry ensures a conformal fit, improving sensor accuracy and patient compliance for personalized products [143]. In regenerative medicine, 3D‐printed hydrogels are being explored for cardiac patches using stem cell therapy as a therapeutic method for the treatment of ischemic heart diseases [144]. Despite its many advantages, 3D printing of hydrogel patches faces challenges such as maintaining cell viability during printing, achieving sufficient mechanical strength for handling, and scaling up production for clinical use [145, 146, 147]. Researchers are addressing these limitations through innovations in bioink formulations, such as nanocomposite hydrogels reinforced with nanoparticles for enhanced durability, or hybrid systems combining multiple polymers to optimize printability and function [148]. Additionally, advancements in printing technologies are enabling faster production speeds and higher resolution, paving the way for more complex and functional designs.

The majorly used 3D printing technologies for hydrogel patch fabrication are:

### 3.2 Extrusion‐Based Printing (Direct Ink Writing)

Extrusion‐based printing, also known as the Direct Ink Writing (DIW) technique is used to fabricate hydrogel patches by depositing shear‐thinning bioinks layer‐by‐layer through a nozzle. This technique relies on hydrogels that exhibit shear‐thinning behavior, allowing smooth extrusion under pressure and rapid shape retention post‐deposition [149]. Commonly used materials include alginate, GelMA, and hyaluronic acid, which can be crosslinked via ionic, photo‐, or thermal techniques to stabilize the printed structure [150]. DIW enables precise control over patch architecture, pore size, and mechanical properties, making it ideal for numerous medical applications [151]. A key advantage of DIW is its ability to incorporate cells and bioactive molecules during printing, facilitating the development of functional, cell‐laden patches. Additionally, DIW supports multimaterial printing, allowing gradients in composition or stiffness within a single patch. However, the technique faces challenges, including limited resolution compared to photopolymerization techniques and the need for optimized ink rheology to prevent clogging or deformation [152]. Cell viability can also be compromised by shear stress during extrusion. Regardless, DIW remains a widely used approach due to its cost‐effectiveness, scalability, and compatibility with a broad range of hydrogel formulations, making it a promising tool for advancing hydrogel patches for biomedical applications.

### 3.3 Stereolithography and Digital Light Processing

Stereolithography (SLA) and Digital Light Processing (DLP) are high‐resolution 3D printing techniques that fabricate hydrogel patches through layer‐by‐layer photopolymerization of liquid resin using UV light. In SLA, a laser precisely cures the hydrogel precursor in a vat, while DLP projects entire layers simultaneously through a digital mask, enabling faster printing [153]. Both techniques use photocrosslinkable hydrogels like GelMA, PEGDA, or hyaluronic acid derivatives, which solidify upon UV exposure to form intricate, water‐swollen networks [154, 155]. These techniques excel in producing hydrogel patches with exceptional precision (10–100 µm resolution), smooth surfaces, and complex geometries, making them ideal for applications like scaffolds for tissue engineering, drug delivery, and wound healing [156]. These techniques can be used to create microarchitectures that mimic natural tissues, such as vascular networks or porous scaffolds for cell infiltration. Additionally, SLA/DLP allows multi‐material printing by switching resins, enabling spatially controlled mechanical or biochemical properties. However, challenges include limited material choices, as only UV‐curable hydrogels can be used, and potential cytotoxicity from photoinitiators. Oxygen inhibition can hinder curing near surfaces, affecting patch stability. Post‐processing, such as washing uncured resin, is often required. Despite these limitations, SLA/DLP remain powerful tools for fabricating advanced hydrogel patches with high fidelity and functionality, driving innovations in biomedical materials/devices.

### 3.4 Inkjet Printing (Drop‐on‐Demand)

Inkjet printing is a precise technique for fabricating hydrogel patches by depositing tiny droplets of bioink in a controlled, layer‐by‐layer manner. This technique relies on thermal or piezoelectric actuators to eject hydrogel precursors, such as alginate, gelatin, or polyethylene glycol (PEG), onto a substrate with high spatial resolution [157]. The process enables the creation of complex, customizable hydrogel structures with embedded cells, drugs, or growth factors, making it ideal for biomedical applications. This technique upholds a high resolution (down to ∼50 µm), allowing for fine‐tuned architectures that mimic natural tissue. It also provides a non‐contact, low‐waste process, reducing material loss and enabling scalable production. The ability to print multiple bioinks simultaneously further enhances functionality, such as gradient hydrogels with varying mechanical or biochemical properties [158]. However, typical shortcomings like the need for low‐viscosity bioinks (<20 mPa s) to ensure smooth droplet formation often limit material choices. Moreover, cell viability may be affected by shear stress during droplet ejection, requiring careful optimization of printing parameters. Clogging of nozzles due to hydrogel aggregation or cell sedimentation could be another limitation, necessitating frequent maintenance. Despite these challenges, inkjet printing remains a promising approach for producing advanced hydrogel patches with high precision and versatility, paving the way for tailored biomedical solutions [159].

### 3.5 Laser‐Assisted Bioprinting

In the Laser‐Assisted Bioprinting (LAB) technique, a pulsed laser beam interacts with a donor layer coated with hydrogel bioink, generating vapor bubbles that propel droplets onto a substrate in a layer‐by‐layer fashion. This non‐contact technique allows for the printing of delicate, cell‐laden hydrogels, such as gelatin or fibrin, with micrometer‐scale resolution while maintaining high cell survival rates [160]. The LAB approach can handle high‐viscosity bioinks without clogging, enabling the incorporation of dense cell suspensions or extracellular matrix components. LAB provides exceptional spatial control, making it ideal for creating complex, multicellular architectures that mimic native tissues, such as skin grafts or vascularized patches [161]. However, LAB is often limited by scalability due to its relatively slow printing speed and high costs associated with laser systems. The technique requires careful optimization of laser parameters (e.g., wavelength, pulse duration) to prevent cell damage, and the choice of bioink must balance printability with biocompatibility. Nevertheless, LAB techniques remain a powerful tool for producing hydrogel patches with precise cellular organization, holding great potential in biomedicine and therapeutic applications.

Printing technologies are still being developed. Therefore, the potential applications of 3D‐printed hydrogels will expand, driving innovation in healthcare and improving patient outcomes. It is anticipated that future research will focus on refining bioink properties, optimizing printing processes, and integrating smart functionalities to create next‐generation patches that seamlessly interact with biological systems.

### 3.6 Microfluidic Fabrication

The microfluidic technique is a powerful tool for constructing hydrogel patches with precise control over their size, shape, composition, and functionality [164]. This technique leverages fluid manipulation at the microscale (typically channels with dimensions of tens to hundreds of micrometers) to create highly tunable hydrogel structures [165]. Hydrogel patches fabricated via different microfluidics techniques (Table 3) find applications in drug delivery, wound healing, tissue engineering, and wearable biosensors [166, 167, 168]. The microfluidic hydrogel patch fabrication technique relies on laminar flow (low Reynolds number, *Re* << 1), which ensures predictable fluid behavior; droplet‐based or continuous‐flow systems for hydrogel formation; crosslinking mechanisms (photo‐polymerization, ionic crosslinking, thermal gelation, or enzymatic reactions); and precision in geometry that is controlled by channel design and flow rates (Figure 5B) [169, 170, 171]. Microfluidic fabrication enables the production of hydrogel patches with precise architectures and functionalities, making them ideal for biomedical applications; however, they are seldom challenged by blockage of microchannels by polymer aggregates, scaling up while maintaining uniformity is difficult, and biocompatibility issues to ensure non‐toxic crosslinking techniques [172]. Integrating microfluidics with 3D bioprinting techniques [173] and ultrasound [174] can overcome these challenges and prepare complex hydrogel patches with high material encapsulation efficiency.

**TABLE 3:** Different microfluidic techniques for the fabrication of functional hydrogel patches and their applications.

Type of microfluidic	Key principle	Advantages	Challenges	Typical example			Ref.
				Precursor materials	Application	Findings	
Droplet‐based microfluidics	Generating monodisperse droplets of hydrogel precursors in an immiscible carrier fluid, followed by gelation to form solid microgels or patches	Monodispersity, high throughput, versatility, scalability	Clogging, limited patch size, biocompatibility	Polydimethylsiloxane (PDMS) molds	Wound management	Stable adhesion during the long‐term recovery process of chronic wounds	[175]
				Ca‐alginate hydrogel	Drug delivery	Dermal patch with integrated flexible heater	[176]
Continuous‐flow lithography	Combines photolithography with microfluidics, allowing for the rapid polymerization of hydrogel structures in a flowing stream	High throughput, precision patterning, multimaterial integration, cell encapsulation, scalability	Oxygen inhibition, throughput vs. resolution trade‐off, material constraints	Poly(ethylene glycol) diacrylate (PEGDA) and polydimethylsiloxane (PDMS)	Anticounterfeiting	Soft matter‐inspired product quality control, tracking, and anti‐counterfeiting technologies	[177]
				Poly(hydroxyethyl methacrylate)	Biosensors	Sweat sensing reliability without sebum interference	[28]
Microfluidic spinning	Leverages microfluidic devices to extrude hydrogel precursors in a controlled manner, followed by in situ crosslinking to form continuous or patterned structures	Tunable fiber diameter (10–500 µm), high cell encapsulation, multimaterial	Particle aggregation in microchannels, slower than bulk techniques, hydrogels may be too soft for some applications	Prolamins‐assembled porous hydrogel microfibers	Wound healing	Superior in vivo capability in treating diabetic wounds	[178]
				Na‐alginate, GelMA	Wound repair	Promising stem cell carrier.	[179]
Microfluidic electrospray	Combines microfluidics with electrospraying to produce hydrogel patches with controlled morphology, high encapsulation efficiency, and tunable release properties	>90% encapsulation efficiency, monodisperse particles with narrow size distribution, low shear stress, precision patterning	Clogging, slower than bulk techniques, optimization of the electric field, and crosslinking for sensitive cells	Chitosan oligosaccharide‐polyacrylic acid	Wound closure and healing	Adhesive patch for wound management and battlefield nursing	[180]
				Microalgae‐loaded living alginate	Tissue repair	Promotes collagen deposition and vascular generation during the wound closure processes	[181]
Slab hydrogel patterning	used to create spatially customizable hydrogel structures with defined geometries, chemical gradients, and microarchitectures	High spatial resolution (µm‐scale features), customizable biochemical and mechanical gradients, compatibility with cell encapsulation, and scalable for large‐area patches	Clogging in microchannels (due to high‐viscosity precursors), limited thickness control	Lignin‐polyacrylamide	Soft electronics	Microfluidic‐assisted hydrogel patches for monitoring health under extreme climates (e.g., rescue operations in polar regions)	[182]
				3D origami MN patch printed with MXene electrocircuits	Wound management	Detection of biomarkers, controlled drug release, and motion monitoring for wound healing	[183]
Microfluidic flow‐cytometric	Leverages droplet microfluidics and fluorescence‐activated sorting (FACS‐like mechanisms) to generate highly uniform hydrogel microbeads or patches	High uniformity, single‐cell precision, scalability, multifunctionality	Throughput limitations, clogging risks, biocompatibility	PDMS, PEGDA	Counting residual white blood cells (rWBC)	Integration of target cell enrichment, on‐chip cell staining capabilities, enables high‐throughput, sensitive, and sample preparation‐free rWBC counting.	[184]

## 4 Biomedical Applications

In addition to their experimental versatility, several epidermal patch technologies have demonstrated clinically relevant, preclinical, or commercially validated performance across drug delivery, biosensing, wound care, and therapeutic monitoring applications. Representative examples are highlighted below to emphasize the translational potential of these systems.

### 4.1 Drug Delivery

**FIGURE 6:**
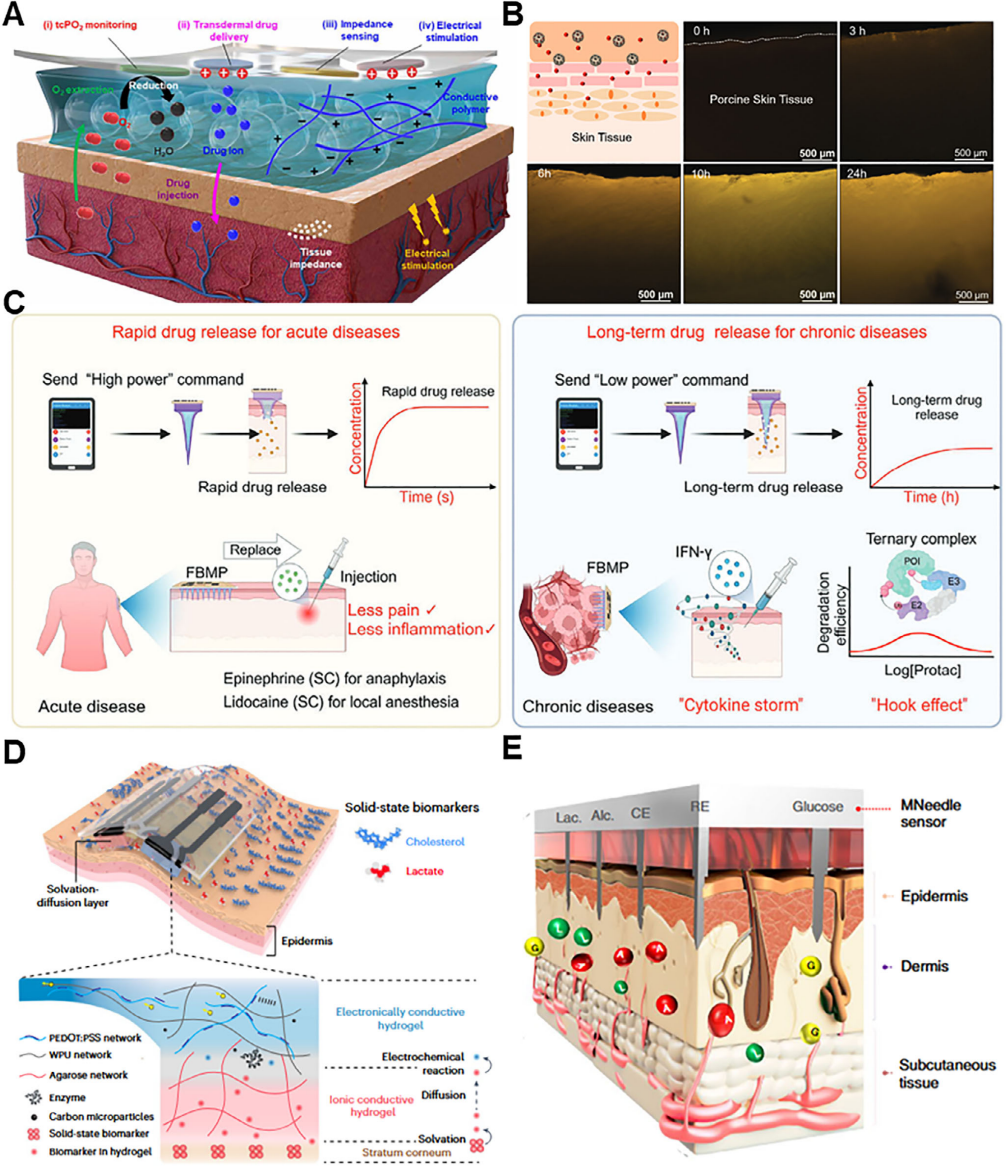
(A) Illustration of the structure, requirement, and roles of the hydrogel interface between human skin and the wearable bioelectronics. The exemplified wearable device is used for transcutaneous bioanalysis and therapy, such as transdermal drug delivery, tissue impedance sensing, and electrical stimulation. (Reproduced with permission [186]. Copyright 2021, American Association for the Advancement of Science) (B) Tissue penetration profile of the released R6G from PAM/PDA/XL‐MSN(R6G) hydrogel through the porcine skin according to attaching time. (Reproduced with permission [187]. Copyright 2020, Wiley‐VCH) (C) The application of a patch for rapid drug release and long‐term drug release. (Reproduced with permission [190]. Copyright 2025, Wiley‐VCH) (D) Illustration of the SEB sensor on the skin and schematics showing the cross‐section of the SEB sensor, displaying the ICH, ECH, and stratum corneum, and the components in each hydrogel layer. (Reproduced with permission [202]. Copyright 2024, Springer Nature. (E) A cross‐sectional illustration of the sensor patch with the MNs piercing the skin to reach the epidermis. Reproduced with permission [204]. Copyright 2022, Springer Nature.

Drug delivery technology plays a crucial role in modern medicine, aiming to optimize therapeutic outcomes and minimize side effects by refining drug release mechanisms [185]. Hydrogel‐based drug delivery systems have emerged as a significant category within this field. Lim et al. designed an ultra‐soft, mass‐permeable, and low‐impedance hydrogel interface that achieves efficient drug delivery through seamless skin contact [186]. The ultra‐thin thickness and porous structure of this hydrogel significantly enhance the diffusion efficiency of drug molecules. Additionally, the incorporation of conductive polymers such as PEDOT: PSS reduces interfacial impedance, making it suitable for electrostimulation and drug delivery. This technology not only facilitates precise drug delivery through electrostimulation but also enables real‐time physiological monitoring, offering new solutions for epidermal patches in the medical field (Figure 6A). In another study, Jung et al. developed a hydrogel patch based on polyacrylamide (PAM) and polydopamine (PDA), embedded with extra‐large pore mesoporous silica nanoparticles (XL‐MSNs) to significantly enhance adhesion and mechanical strength [187]. This hydrogel patch achieves passive drug release through the diffusion mechanism, leveraging the covalent bonding and physical interactions of PDA chains with skin surface groups. The penetration of the rhodamine 6G (R6G) released into the tissue was analyzed by observing the cross‐section of the skin tissue under a fluorescent microscope (Figure 6B). In contrast, Zhou et al. introduced an electro‐responsive hydrogel (ERH) that utilizes direct current voltage (DCV) to reorganize sodium dodecyl sulfate (SDS) micelles, thereby adjusting the hydrogel network's pore size for precise drug release [188]. This hydrogel stands out with its high stretchability (>6000%) and toughness with a high fracture energy (507 Jm^−2^), as well as its self‐healing and malleable properties, making it suitable for smart drug delivery systems and wearable devices. The evolution from passive to electro‐controlled release in hydrogel technology represents a notable leap in precision.

On the other hand, MN‐based drug delivery technologies have revolutionized transdermal delivery by overcoming skin barriers and enhancing drug transport efficiency [189]. Jin et al. presented a flexible bioelectronic MN patch (FBMP) that integrates flexible electronics and MN technology, with drug release controlled via Bluetooth‐connected heating films [190]. This system employs a thermal response mechanism to accelerate drug release and uses a bilayer MN structure to penetrate the stratum corneum, creating microchannels for efficient drug transport (Figure 6C). In addition, dos Santos et al. developed eutectogel MN patches (EU‐MNs) that address the solubility challenges of hydrophobic drugs using natural deep eutectic solvents (NADES) [191]. The EU‐MNs, built on a GelMA/PEGDA matrix, ensure sustained drug release over several days. The dual‐network architecture of these MNs offers adequate mechanical strength for skin penetration while maintaining structural integrity, expanding the prospects for tailored medical applications in hydrophobic drug delivery.

Hydrogel‐based systems are at the forefront of these developments, offering unique advantages such as efficient drug release, real‐time physiological monitoring, and enhanced adhesion. As research progresses, the convergence of these technologies could lead to even more advanced systems that combine the benefits of hydrogels and MNs, offering both precise control and enhanced patient comfort. The development of such systems could revolutionize medicine by enabling personalized treatment plans, improving patient compliance, and reducing the risk of side effects. The potential applications of these technologies are vast, ranging from chronic disease management to post‐operative care, and their continued evolution will likely have a profound impact on the future of healthcare.

Collectively, these examples show that epidermal patch‐based drug delivery systems are moving beyond proof‐of‐concept designs toward clinically relevant platforms with controllable release profiles, improved tissue penetration, and enhanced patient usability.

### 4.2 Health Monitoring

Advancing from surface monitoring, hydrogel‐based epidermal patches offering enhanced comfort and biocompatibility for health monitoring [66, 87]. As shown in Figure 6D, Arwani et al. designed a stretchable ionic‐electronic bilayer hydrogel sensor that enables the in situ detection of solid‐state epidermal biomarkers (SEB) [192]. This sensor uses a bilayer hydrogel structure to facilitate the solvation, diffusion, and electrochemical reaction of solid‐state analytes, providing ultra‐low detection limits (0.51 nmol cm^−2^ solid lactate and 0.26 nmol cm^−2^ solid cholesterol). Similarly, Sempionatto et al. developed a non‐invasive skin patch that monitors hemodynamic and metabolic biomarkers simultaneously [193]. This patch uses ultrasonic sensors to measure blood pressure and heart rate by analyzing the time of flight of echoes from arterial walls. Meanwhile, electrochemical sensors detect biomarkers like glucose, lactate, caffeine, and alcohol in sweat and ISF through enzymatic reactions. The device can capture physiological changes caused by activities such as food intake and exercise.

Moreover, Tehrani et al. created an integrated wearable MN array that continuously monitors multiple biomarkers in ISF Figure 6E [194]. This array uses MNs to access ISF and detect substances like lactate, glucose, and alcohol through electrochemical reactions, with data transmitted wirelessly to a smartphone app for real‐time analysis. Further expanding the scope of health monitoring, Lu et al. developed a stretchable graphene‐hydrogel interface for wearable epidermal patches and implantable bioelectronics [195]. Created using cryogenic transfer techniques, this interface allows for the monitoring of various physical and physiological signals, including mechanical deformation, temperature, humidity, and ECG signals, both on the skin and within the body. Another recent work presents a wearable microneedle (MN) sensor patch for diabetes monitoring [196]. The patch uses a dual‐crosslinking hydrogel matrix of methacrylic acid‐modified gelatin and polyvinyl alcohol (PVA) to boost both mechanical strength and swelling capacity for efficient interstitial fluid (ISF) uptake. The MN array is integrated with enzyme‐ and chromogenic dye‐functionalized test strips, realizing in vivo colorimetric detection of multiple diabetes‐associated biomarkers such as glucose and ketone bodies. These innovations represent a progression from single‐function devices to multi‐functional integration, significantly enhancing the depth and application range of health monitoring technologies.

As research continues to advance, the potential for these technologies to revolutionize personalized healthcare becomes increasingly evident. Imagine a future where these patches could integrate with AI‐driven platforms to provide predictive health analytics, allowing for early intervention and prevention of health issues before they become critical. The seamless combination of various sensing modalities could also pave the way for holistic health monitoring, where physical, biochemical, and environmental data are collected and analyzed in tandem. The development of such technologies could democratize healthcare by making advanced diagnostic tools more accessible and user‐friendly. As these hydrogel‐based systems continue to evolve, they may not only transform how we monitor health but also redefine the boundaries between the human body and technological interfaces, ultimately leading to more proactive and personalized approaches to wellness and disease management. These examples demonstrate that epidermal patch technologies are increasingly aligned with real‐world healthcare needs by enabling continuous, user‐friendly, and multimodal physiological monitoring outside conventional clinical settings.

### 4.3 Hydrogel Micropatch Sampling Probes for Skin Metabolomics

Metabolomics identifies and measures metabolite pools that underlie an organism's structure, function, and dynamics [197]. Thanks to technological developments, numerous metabolites can be quantitatively measured in small amounts of biological material, enabling systems‐level analyses [198]. Metabolomics is used as a tool for biomarker discovery [199]. The research in this field heavily relies on analytical instrumentation, in particular, mass spectrometry (MS) [200] – a technique that provides high sensitivity [201] and quantitative capabilities [202]. One of the key steps in MS is the ionization of molecules in the gas phase. Electrospray ionization (ESI) [203] is among the most useful ionization techniques for metabolomic studies. In ESI, a liquid sample is infused through a capillary supplied with a high voltage. In the 21st century, a number of techniques have emerged (in some cases, through modification of ESI), which enable direct analysis of solid matrices (e.g., ref. [204]). For example, in nanoDESI, a minute amount of solvent is relayed onto the sample surface to extract analytes, and the extract is directed toward a mass spectrometer's orifice through a short capillary section [205]. Alternatively, a specially designed tubing system (liquid microjunction surface sampling probe or LMJ‐SSP) can be used to extract analytes from the solid sample and to transfer them to the ESI source [206, 207]. In the past few decades, much of the attention has been devoted to analyzing conventional biological specimens such as blood and urine. However, more recently, researchers also considered alternative matrices such as hair, tears, saliva, and skin excretions [208]. In many aspects, the collection of such specimens is more convenient than the collection of conventional specimens. A number of studies have been carried out to investigate skin metabolomes [209].

**FIGURE 7:**
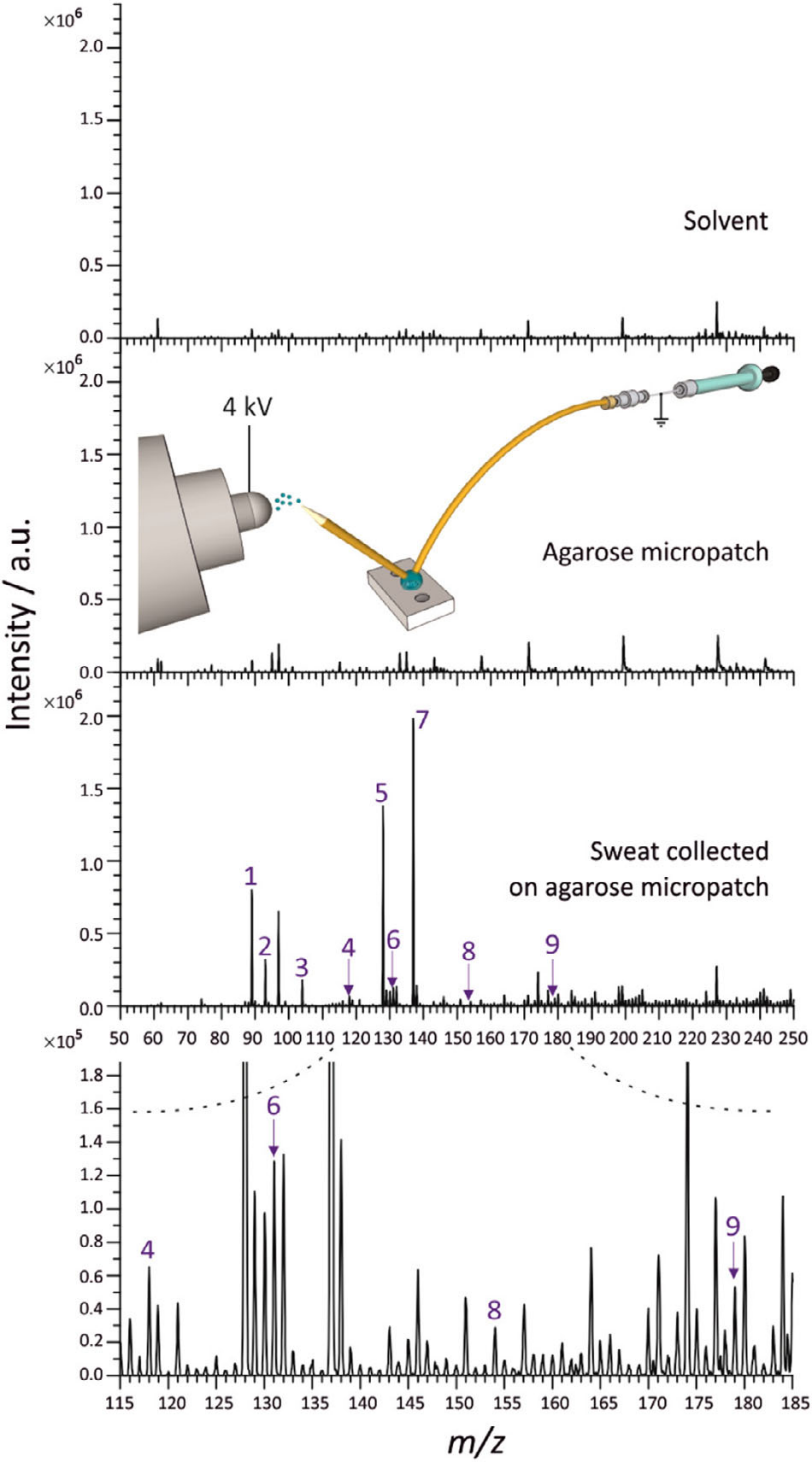
Mass spectrometric profiling of hydrogel‐trapped metabolites. The upper spectra correspond to blanks (the solvent and freshly made hydrogel probe). The lower part shows the result of sweat analysis using the proposed approach. Sample collection time: 3 h. Analysis time: 2 min. Solvent: acetonitrile: water (9:1, v/v) spiked with ammonium hydroxide (final concentration of 0.1%). The inset shows the setup for analysis, incorporating a nanospray desorption ionization [205] interface directly united with the hydrogel micropatch probe via the solvent bridge. Numbers (1–9) indicate the identified peaks: (1) lactic acid; (2) fragment of urocanic acid; (3) serine; (4) threonine; (5) pyroglutamic acid; (6) ornithine; (7) urocanic acid; (8) histidine; and (9) paraxanthine. Reprinted with permission from [213]. Copyright 2014 American Chemical Society.

In principle, skin metabolites can be sampled using simple and widely available tools such as polyester swabs [210]. However, efforts were made to produce other specialized sampling tools addressing application‐specific requirements. For example, Chen et al. recently developed a standard operating procedure for the investigation of skin surface metabolites, which is based on the use of a special tape [211]. It has also been known that hydrogel‐based materials can be useful for testing skin pathology [212]. In 2014, Dutkiewicz et al. presented a hydrogel micopatch method for sampling skin metabolites [213, 214]. Agarose hydrogel micropatches were embedded in polytetrafluoroethylene plates. The resulting probes are affixed onto the skin to transfer metabolites from the skin to the hydrogel micropatches. Subsequently, the hydrogels are re‐extracted by a solvent in the nanoDESI interface, and the metabolites are analyzed by MS (Figure 7). This method enabled analysis of low‐molecular‐weight compounds present in the skin of healthy volunteers. Furthermore, it was applied in a study involving patients suffering from psoriasis and healthy individuals [215]. Several putative skin biomarkers of psoriasis have been discovered. Later on, the method was used to track the progress of recovery in response to treatment with biologics by analyzing skin metabolites [216]. Interestingly, it was found that blood metabolite concentrations did not correlate with skin metabolite concentrations.

In other work, it was suggested that analyzing compounds, such as tryptophan and its metabolite kynurenine, may offer a non‐invasive strategy for detecting skin cancer [217]. An in vitro pig skin permeability study—performed by Jankovskaja et al.—demonstrates that non‐invasive topical sampling of tryptophan and kynurenine is feasible, pointing to the potential of the tryptophan/kynurenine ratio as a skin cancer biomarker [218]. Later, Jankovskaja et al. examined various topical sampling techniques for tryptophan and kynurenine, compared with phenylalanine and tyrosine, on the volar forearms of healthy individuals [217]. The methods involved three types of hydrogels (made from agarose and/or chitosan), hydrated starch films, cotton swabs, and tape stripping. In a related work, Morin et al. further explored the use of hydrogels (chitosan and agarose) and cubic liquid crystals (based on glycerol monooleate) for non‐invasive sampling of low‐molecular‐weight biomarkers [219]. Notably, the cubic phases demonstrated an extraction capacity about twice as high as that of the hydrogels. In the studies involving human subjects, liquid chromatography (LC)‐MS was used for metabolite quantification.

A disadvantage of using agarose‐based hydrogels for sampling skin metabolites is that such probes are vulnerable to drying. Therefore, they have to be prepared shortly before sampling, and analyzed within hours after sampling. Thus, there is a need to search for other hydrogel variants for sampling skin excretions. An attempt was made to modify the composition of the hydrogel to make the hydrogel probes rugged for sampling in the clinical environment, and the sampling was performed by LMJ‐SSP [220]. The hydrogel micropatch probes have evolved to micropatch arrayed pads, which enable mapping of chemical species on skin by means of nanoDESI‐MS [221]. Blended hydrogel micropatches have also been used to collect analytes from other surfaces than skin, and a method called “gel‐phase microextraction” has been proposed [222]. The technique was further modified to incorporate enzymatic reactions into hydrogel micropatches and enable mapping of glucose in food‐related specimens [223]. However, those mapping biosensors are not currently available for mapping skin metabolites due to the toxicity of some reactants embedded in hydrogels. Nevertheless, it may be possible to transfer metabolites from the skin surface onto pure hydrogel slabs and then perform mapping metabolites on those slabs. There has also been an attempt to implement a miniature robotic arm for sampling skin metabolites using a hydrogel prior to extraction MS analysis [224].

To sum up, several studies have demonstrated the usefulness of hydrogels and related materials for sampling skin metabolites. Those sampling probes are compatible with MS‐based methods, which rely either on online re‐extraction and direct infusion or off‐line sample treatment and LC‐MS analysis. This technology is promising for biomarker discovery of skin‐related and systemic diseases. Since a swellable MN patch can be used to sample skin ISF [225], it is appealing to couple those patches with the MS interfaces described above to enable the detection of biomarkers in ISF.

### 4.4 Tissue Engineering

**FIGURE 8:**
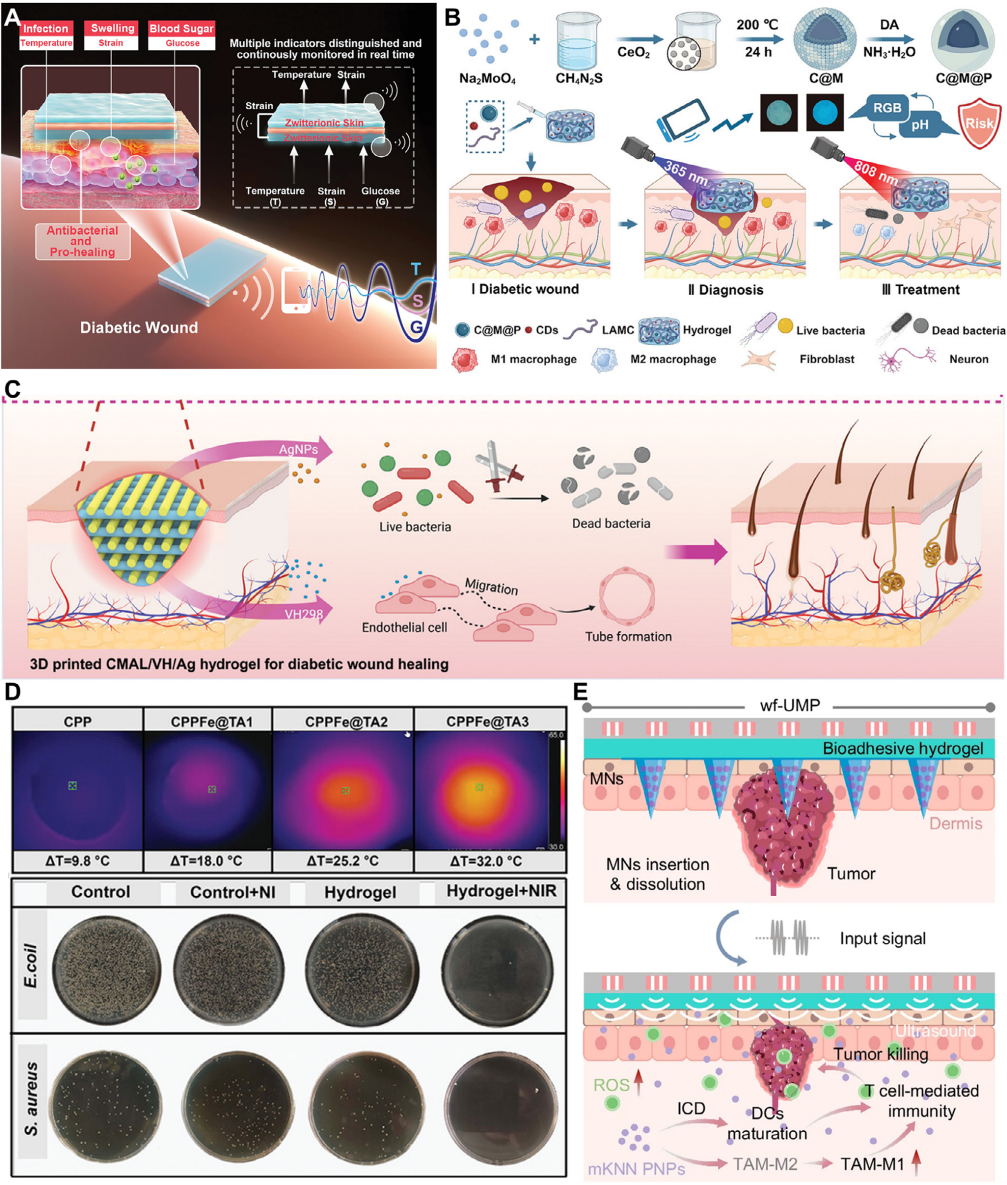
(A) Scheme illustration of the sandwich‐structured sensor based on multi‐response zwitterionic skin for multiple sensations and pro‐healing of diabetic wounds. (Reproduced with permission [226]. Copyright 2021, Wiley‐VCH) (B) Schematic illustration of constructing a diagnostic and therapeutic hydrogel for diabetic wound healing. (Reproduced with permission [227]. Copyright 2024, Wiley‐VCH) (C) Diagram for the regenerative application of the high self‐supporting CMAL hydrogel ink. (Reproduced with permission [228]. Copyright 2025, Wiley‐VCH) (D) Photograph of the antibacterial effect of the hydrogels against *E. coli* and *S. aureus*. (Reproduced with permission [229]. Copyright 2024, Wiley‐VCH) (E) Schematic illustration of the operational procedure of wf‐UMP for tumor immunotherapy. (Reproduced with permission [230]. Copyright 2025, Wiley‐VCH).

In the field of chronic wound management, ​Guo et al. developed a sandwich‐structured sensor system based on a zwitterionic thermo‐glucose‐sensitive hydrogel [226]. By synergistically decoupling capacitive and resistive signals, this system achieved interference‐free real‐time monitoring of temperature, strain, and glucose concentration (Figure 8A). Its unique anti‐protein adsorption properties simultaneously created an antibacterial microenvironment for diabetic ulcers. This work inspired subsequent research toward integrated diagnosis and therapy. For example, ​Wang et al. proposed a multifunctional hydrogel based on lipoic acid‐modified chitosan (LAMC) hydrogel, carbon quantum dots (CDs), and ceria oxide‐molybdenum disulfide nanoparticles with polydopamine (C@M@P). The developed hydrogel (LAMC/CD‐C@M@P) combined diagnostic and therapeutic functions for intelligent monitoring and treatment of diabetic wounds [227]. The CDs embedded in the hydrogel provided high sensitivity and reversibility to pH changes, enabling real‐time monitoring of wound pH through fluorescence intensity changes, providing a basis for early detection of bacterial infections (Figure 8B). Moreover, the C@M@P in the hydrogel exhibited excellent photothermal antibacterial properties under near‐infrared (NIR) irradiation and the ability to scavenge ROS, alleviating oxidative stress and inflammatory responses. In a diabetic mouse model, this hydrogel achieved a 95.49% wound healing rate, marking the integration of smartphone RGB analysis technology into intelligent wound management.

For regenerative applications, Li et al. developed a high self‐supporting chitosan‐based hydrogel ink (CMAL) for in situ 3D printing of diabetic wounds. By introducing nanoclay (LAP) and amide bonds, the hydrogel forms a dense physical cross‐linking network, achieving high‐precision 3D printing [228]. The silver nanoparticles (AgNPs) and hypoxia‐inducible factor‐1α (HIF‐1α) stabilizer (VH298) incorporated in the hydrogel provide antibacterial and angiogenic functions, respectively. In a mouse model of diabetic wounds, the 3D printed hydrogel significantly accelerated wound closure and promoted collagen deposition and the formation of new blood vessels (Figure 8C). On the other hand, ​Dang et al. designed an injectable hydrogel using carboxymethyl cellulose (CMC) and tannic acid/Fe^3+^ complexes [229]. By regulating crosslinking time through Fe^3+^ concentration, they achieved 99% sterilization efficiency via NIR photothermal effects, while the ionic conductivity of CMC enabled physiological signal monitoring, providing a dynamic microenvironment for tissue regeneration. Compared to the blank control group, the survival rates of bacteria significantly decreased, which could be attributed to the destructive effect of abundant catechol groups derived from tannic acid in the hydrogel (Figure 8D).

Furthermore, Xue et al. designed a flexible, wearable ultrasound microneedle patch (wf‐UMP) that provides a portable platform for cancer therapy (Figure 8E) [230]. The wf‐UMP integrates a stretchable, lead‐free ultrasound transducer array (stable under 60% tensile strain) for acoustic emission, a bioadhesive hydrogel elastomer (interfacial toughness > 500 Jm^−2^ on skin) that ensures both skin adhesion and acoustic coupling, and a dissolvable MN patch embedded with biocompatible piezoelectric nanoparticles for non‐invasive (penetration depth up to 500 µm in porcine skin) drug delivery and reactive oxygen species (ROS) generation. The device exhibited potent anticancer activity by inducing apoptosis in tumor cells, enhancing oxidative stress, and modulating immune cell proliferation. The synergistic immunotherapy achieved through the combined action of the wf‐UMP and Anti‐PD1 enhanced anticancer immunity by promoting immunogenic cell death and regulating macrophage polarization, thereby suppressing distant tumor growth and recurrence. In preclinical mouse studies, the wf‐UMP exhibited markedly superior efficacy in delaying and suppressing tumor growth compared to control groups, as reflected by significantly reduced tumor weights and volumes. Transcriptomic analysis further revealed upregulation of cytokine–cytokine receptor interactions and T cell receptor signaling pathways following wf‐UMP treatment, underscoring its capacity to enhance antitumor immune responses. The device's secure conformability to curved and dynamic tissue surfaces, coupled with efficient acoustic emission and improved drug delivery performance, highlights its promise as a versatile platform for cancer immunotherapy.

The ongoing innovation in hydrogel technology is reshaping the landscape of tissue engineering, such as wound management and cancer therapy, by merging advanced material science with clinical needs. These innovative hydrogels herald a shift toward proactive healthcare, integrating continuous monitoring with therapeutic functionality. Collectively, these studies underscore the evolution from mere wound dressing to intelligent systems that actively participate in the healing process. The incorporation of such advanced features into hydrogels holds great potential to revolutionize wound care through personalized treatment and faster healing. As these technologies mature, they are likely to become integral to both clinical practices and home care settings, transforming how we approach wound management and tissue repair.

#### 4.4.1 Patches for Wound Healing and Treating Acne

While wound healing is a complex process, it can be complicated further by microbial infections. Composite patches are viscoelastic scaffolds with a high liquid‐to‐polymer mass ratio and closely resemble native tissues. For wound healing applications, the high water‐retaining capacity of the hydrogel is a vital factor as large volumes of wound exudate need to be absorbed [231]. Both natural and synthetic polymers and various crosslinking methods have been utilized in the fabrication of composite patches. Current efforts aim to design natural, high‐performance hydrogel composites that improve therapeutic outcomes and mitigate side effects. The use of synthetic substances in patches for wound healing leads to inherent risks such as long‐term toxicity, immune rejection, and potential carcinogenicity. To overcome these issues, patches fabricated using natural and bioactive substances such as resveratrol (from grape seeds), puerarin (from the Chinese medical herb *Radix pueraria*), and curcumin have been developed for the treatment of diabetic wounds [232].

Ding et al. fabricated a natural and multi‐functional medical patch for diabetic wound management by incorporating grape peel, oligomeric proanthocyanidins, and puerarin into carboxymethyl chitosan hydrogel. The formation of hydrogen bonds between the hydroxy groups of polyphenols of proanthocyanidins and puerarin, and the amino groups of carboxymethyl chitosan resulted in high mechanical strength, and favorable photothermal antibacterial properties against *S. aureus* and *E. coli*. The inclusion of grape peel in the patch regulated the immune response by promoting the polarization of M2 macrophages and scavenging the excess ROS. A diabetic mouse wound model was used to validate the therapeutic efficacy of the patch [233].

**FIGURE 9:**
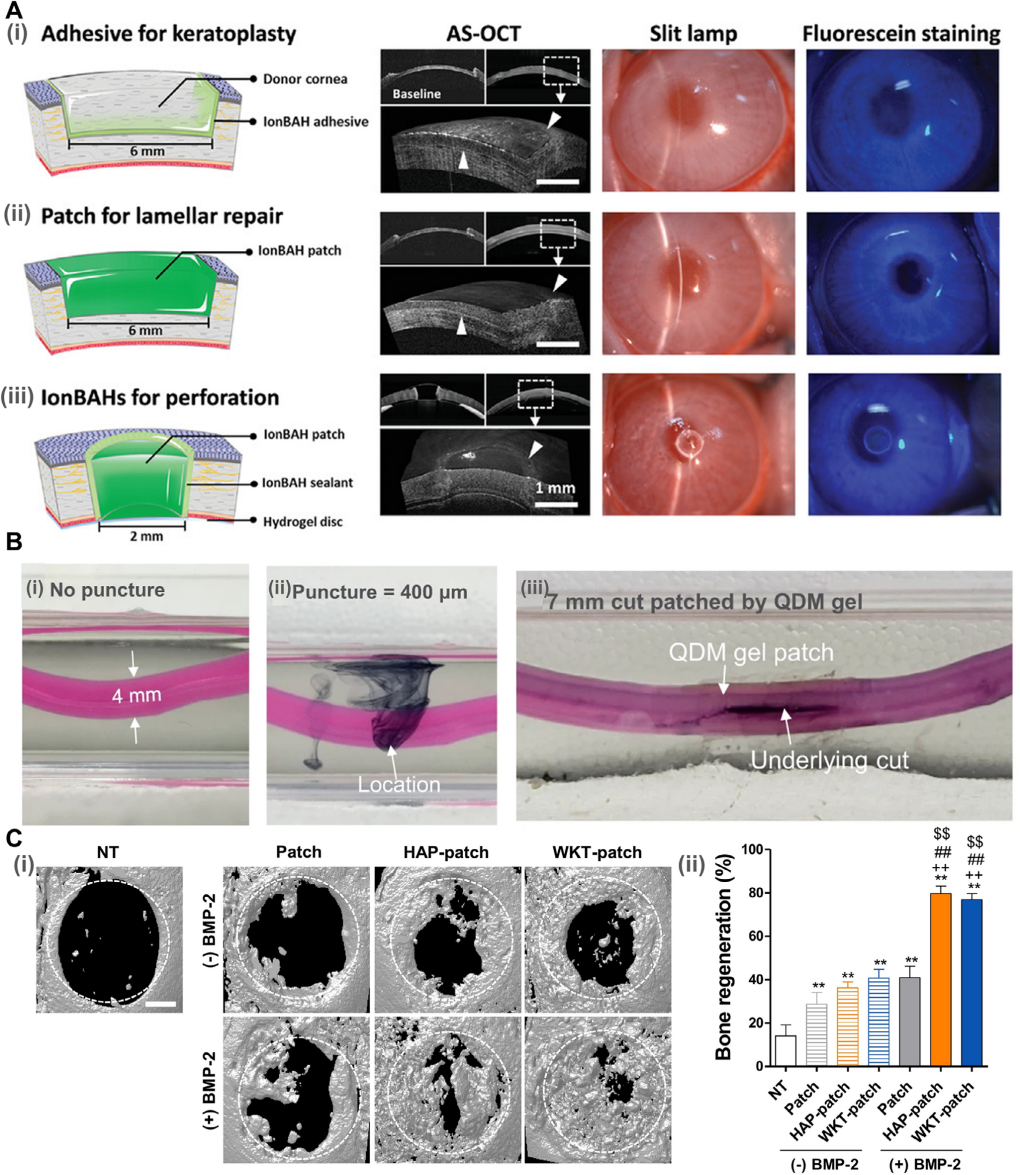
Schematic illustration for the fabrication of a multi‐functional medical patch and its function in wound treatment. (A) Ion‐activated bio‐adhesive hydrogel (IonBH) for repair of large corneal wounds. Image reproduced from ref. [234] with permission from Wiley, 2022. (B) Electro adhesion of a hydrogel based on anionic and cationic monomers showing no leakage when punctured. Image obtained from ref. [235] under Open Access Agreement. (C)(i) Micro‐CT analysis of calvarial defects 8 weeks after implantation of patches. Sale bar = 1 mm. (ii) Quantification of bone regeneration in defective sites treated with patches. Images obtained from ref. [236] with permission from Elsevier, 2020.

Corneal injectable hydrogels are a promising alternative to simplify traditional surgeries and alleviate donor shortages. Zhao et al. developed a novel ion‐activated bio‐adhesive hydrogel (IonBH) for corneal regeneration and repair of large corneal defects of 6 mm [234]. The dual‐network hydrogel comprises native corneal extracellular matrix and peptide‐modified alginate, facilitating transparency, biocompatibility, adhesion, and tunable mechanical properties (Figure 9A). The hydrogel sustains the secretory phenotype of quiescent keratocytes while inhibiting the differentiation of myofibroblasts in vitro. Further, the hydrogel enhanced the fast regeneration of corneal epithelium, stroma, and nerves comparable to the results of donor corneal transplantation. A reversible electroadhesive hydrogel for suture‐less repair of cuts or tears was developed by Borden et al. [235]. The gel patches provided a strong seal over openings in the bovine aorta, and a gel sleeve was able to reconnect segments of damaged gel tubes (Figure 9B). Bio‐inspired adhesive hydrogels have been utilized in cellular and drug delivery systems to tackle diverse tissue diseases. Choi et al. fabricated highly osteoconductive hybrid hydrogel patches by incorporating inorganic minerals, such as hydroxyapatite or whitlockite, into pyrogallol‐conjugated hyaluronic acid. The resulting hydrogels exhibited improved mechanical robustness and distinct structural modifications arising from intermolecular complexation between the inorganic and organic constituents [236]. This intermolecular interaction provided a prolonged and sustained release of the bone morphogenetic protein‐2 (BMP‐2). Improved bone formation by the hybrid patch in a calvarial bone defect was observed (Figure 9C(i) and (ii)).

Using a double network strategy to create multiple non‐covalent interactions, Hao et al. fabricated a paintable composite patch with excellent biocompatibility and antibacterial properties for potential wound dressing [237]. The hydrogel can be conveniently painted to the wound bed as a patch for chronic wound treatment without any adverse exudate leakage. The double network is based on a terpolymer, *N*‐(3,4‐dihydroxyphenylethyl) acrylamide‐acrylic acid‐acrylamide, chitosan, and iron oxide nanoparticles (Fe_3_O_4_). The catechol‐like structures of the terpolymer significantly improved the adhesion strength of the hydrogel to 25.06 kPa, and this was much higher than the commercially available fibrin glue (∼11.5 kPa).

A new type of composite patches was developed for ocular wound healing in large‐sized defects. Open‐globe injuries or full‐thickness wounds cause severe damage to the ocular surface and vision impairment, and advanced surgical procedures are necessary for treating such wounds [238, 239]. In emergency settings, surgical adhesives or patches have found applications in promoting the closure of open‐globe injuries, and products used in the clinics are adhesives such as fibrin glues, cyanoacrylate glues, and polyethylene glycol‐based sealants. Due to their low adhesive strengths (e.g., fibrin glues), release of toxic by‐products (e.g., cyanoacrylate glues), and material degradation (e.g., polyethylene glycol‐based sealants), these types of materials have limited success in the treatment of full‐thickness wounds [240]. The new generation patches have improved mechanical properties and biocompatibility for tissue regeneration.

Jumelle et al. used three photo‐crosslinking polymers, such as GelMA, hyaluronic acid glycidyl methacrylate (HAGM), and PEGDA to fabricate a composite patch with excellent ocular tissue adhesion [241]. Each of the polymers present provided unique properties to the patch so that the patch be conveniently applied beneath a contact lens without the need of any special surgical intervention. GelMA provided excellent adhesion to the ocular tissues, HAGM enhanced the retention of hydrogel prepolymer (initial mixture) on the tissue, and PEGDA provided in vivo biodegradation, flexibility, and stretchability. The ion‐activated composite patch developed by Zhao et al. had desirable transparency, tunable mechanical properties, robust cell adhesion, and excellent biocompatibility [234]. The patch was based on alginate, tetrapeptides (Gly‐Leu‐Lys‐dopamine), and transglutaminases which promoted intermolecular interactions for stable cellular adhesion. The patch was found to rapidly seal large corneal defects, thereby facilitating the formation of the normal curvature of the corneal tissue [239].

Ocular alkali burn injury is an ophthalmic emergency that requires immediate medical attention. Shi et al. developed an electrospun nanofiber patch functionalized with scavengers for ROS for the treatment of ocular alkali burn injuries [120]. The nanofibers were composed of crosslinked polymers, thioketal‐containing polyurethane, and ROS‐scavenging species such as thioketal diamine and 3,3’‐dithiobis (propionohydrazide). Using a rat corneal alkali burn model, the high tensile strength, excellent transparency, reduced inflammation, and accelerated corneal wound healing of the nanofiber patch were demonstrated. Design of ocular patches using predictive modelling that identified the linear and non‐linear correlations between material properties was reported by Gholizadeh et al. [239, 242]. Based on this approach, they developed an adhesive patch through photo crosslinking of two modified polymers, GelMA and HAGM. The patch when applied *ex vivo* on the sclera exhibited high burst pressure, good adhesion, minimal swelling, and prolonged retention.

Electro adhesion is a unique adhesion mechanism in which adhesion between the patch and the tissue is promoted in the presence of an electric field [243]. Borden et al. exploited the principles of electro adhesion and developed an ocular patch based on cationic gels and bovine tissues [235]. The ocular tissue when subjected to an electric field (DC 10 V) for a period of 10 s, promoted good and prolonged adhesion, and upon reversing the polarity the adhesion can be terminated. Such patches provide a robust seal with on‐demand adhesion, overcoming the need for sutures. Another advantage of these patches is, in care of any error, the adhesion can be reversed by simply changing the polarity of the electric field.

## 5 Current Challenges and Future Perspectives

Epidermal patches have emerged as a revolutionary tool in biomedical applications. They offer huge potential in biomedical materials and biosensing tools due to their biocompatibility, tunable mechanical properties, and ability to encapsulate cells and bioactive molecules with remarkable efficiency. However, despite their promise, several challenges hinder their widespread clinical adoption. One major limitation is achieving optimal mechanical strength while maintaining biocompatibility, as many hydrogels are either too fragile for practical use or too rigid to mimic natural tissues. Moreover, ensuring controlled degradation rates that match tissue regeneration timelines remains challenging, with some hydrogels degrading too quickly, losing structural integrity, or persisting too long, causing inflammation. Another critical challenge is the precise spatial distribution of cells and bioactive molecules within the patch, as uneven distribution can lead to ineffective tissue integration or drug release. Scalability and reproducibility in manufacturing also pose significant hurdles, particularly when transitioning from lab‐scale production to clinical‐grade fabrication, where batch‐to‐batch variability must be minimized. Furthermore, sterilizing hydrogel patches without compromising functionality is a persistent issue, as traditional methods like autoclaving or gamma irradiation can alter their physical and chemical properties. In addition, regulatory and standardization challenges slow down commercialization, as hydrogel‐based medical devices often require extensive preclinical and clinical validation to ascertain adequate safety and efficacy. To improve conceptual clarity and avoid fragmentation, the key challenges and their corresponding future strategies are summarized in Table 4.

**TABLE 4:** One‐to‐one correspondence between current challenges and future strategies in hydrogel‐based epidermal patches.

Current challenge	Corresponding strategy/future direction
Mechanical strength vs. biocompatibility trade‐off	Nanocomposite hydrogels reinforced with nanoparticles (e.g., graphene, metal/metal oxides)
Uncontrolled or mismatched degradation rates	Smart, stimuli‐responsive hydrogels with tunable degradation profiles
Uneven distribution of cells and bioactive molecules	3D bioprinting and microfluidic fabrication for spatial precision
Scalability and batch‐to‐batch variability	Automated high‐throughput manufacturing and standardized bioink systems
Sterilization‐induced property degradation	Development of mild or non‐destructive sterilization techniques
Regulatory and standardization barriers	Advanced validation frameworks and standardized testing protocols
Limited adaptability of static systems	4D printing and dynamic, responsive hydrogel platforms
Trial‐and‐error material optimization	Machine learning and computational modeling for predictive design

**FIGURE 10:**
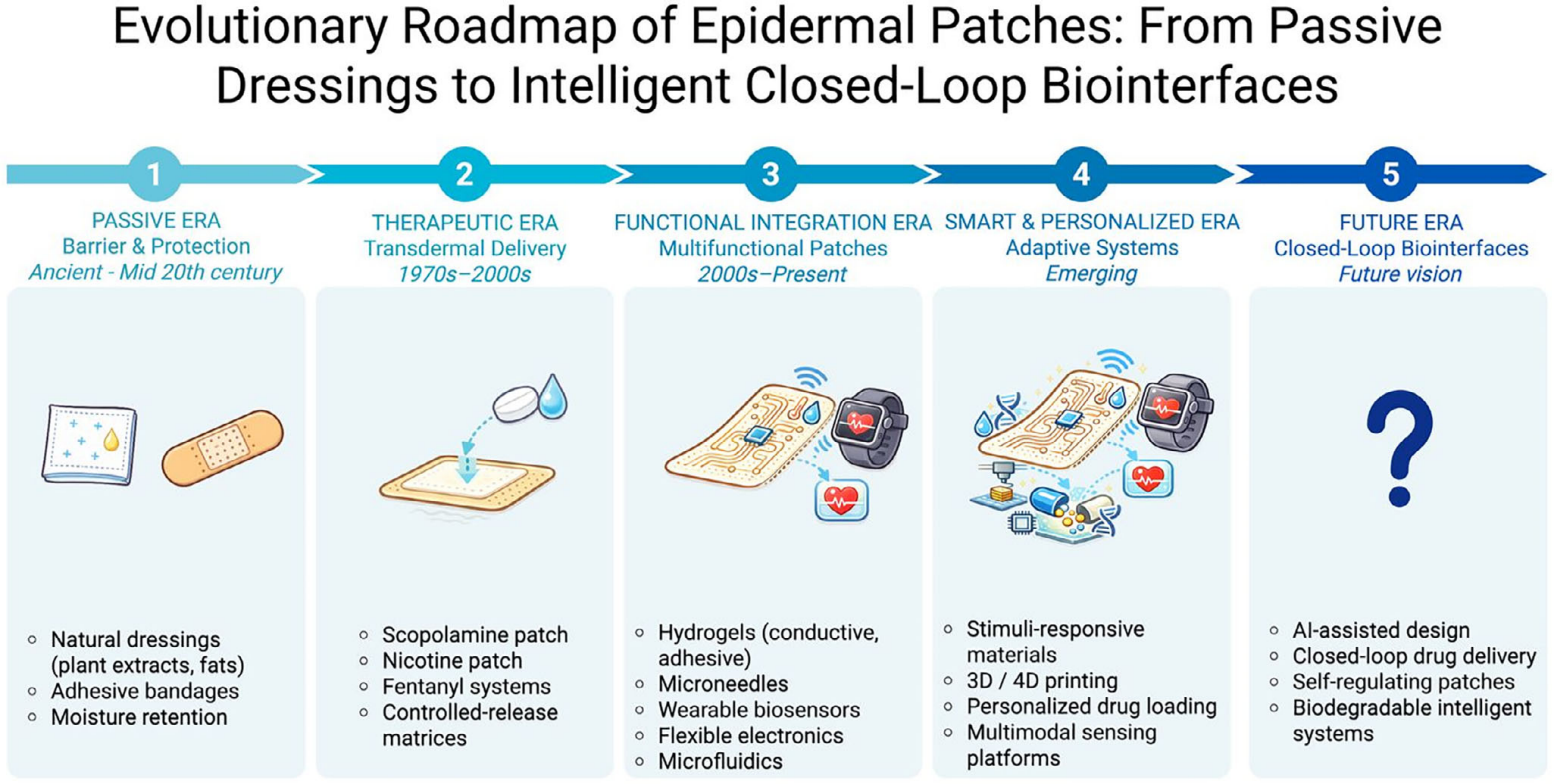
Evolutionary roadmap of epidermal patch technologies. This schematic illustrates the progressive development of epidermal patches across five major eras. The *Passive Era* (ancient to mid‐20th century) is characterized by basic barrier and protective functions using natural dressings and adhesive materials. The *Therapeutic Era* (1970s–2000s) marks the emergence of transdermal drug delivery systems, including scopolamine, nicotine, and fentanyl patches with controlled‐release capabilities. The *Functional Integration Era* (2000s–present) introduces multifunctional platforms incorporating conductive hydrogels, microneedles, wearable biosensors, flexible electronics, and microfluidic systems. The *Smart and Personalized Era* represents the transition toward adaptive systems enabled by stimuli‐responsive materials, 3D/4D printing, and personalized drug loading with multimodal sensing. Finally, the *Future Era* envisions closed‐loop biointerfaces integrating AI‐assisted design, real‐time feedback‐controlled drug delivery, self‐regulating mechanisms, and biodegradable intelligent systems.

Looking into the future, numerous promising strategies are being explored to overcome these limitations. Notably, many of these approaches directly address the specific challenges outlined above, enabling a more systematic transition from fundamental limitations to practical solutions. Advances in 3D bioprinting and microfluidic fabrication techniques enable more precise control over hydrogel architecture, allowing patient‐specific designs with embedded vascular networks to enhance nutrient diffusion and cell survival. Developing smart, stimuli‐responsive hydrogels such as those activated by pH, temperature, or enzymes could improve on‐demand drug release and tissue integration. The construction of nanocomposite hydrogels reinforced with nanoparticles (e.g., graphene, metal/metal oxide nanoparticles) will help to enhance mechanical properties without sacrificing biocompatibility [244, 245]. In perspective, the focus on 4D printing, where hydrogels can dynamically change shape or function in response to environmental signals, could open new possibilities for minimally invasive implantation and adaptive therapies. Furthermore, machine learning and computational modeling tools can be leveraged to optimize hydrogel formulations and predict degradation behavior, reducing trial‐and‐error experimentation [246]. To address scalability, automated high‐throughput manufacturing systems and standardized bioink formulations could be developed to ensure consistency in large‐scale production. The next generation of epidermal patches is being shaped by advances in AI‐driven biosensing, autonomous therapeutic delivery, and self‐healing materials. These platforms aim to provide closed‐loop, intelligent healthcare solutions capable of real‐time monitoring, adaptive drug release, and enhanced tissue regeneration. This evolutionary roadmap highlights a clear trajectory: from passive, single‐function systems to multifunctional, intelligent interfaces that bridge monitoring, therapy, and regenerative applications (Figure 10).

Advances in epidermal patch fabrication technologies, designing innovative materials, and integrating computational design will play a pivotal role in unlocking the full potential of this class of materials and expanding the field of materials science/engineering, thereby paving the way for next‐generation therapies that are minimally invasive and highly effective. As research in this field progresses, epidermal patches may soon become a standard tool in regenerative medicine, drug delivery, and wearable healthcare, transforming biomedicine and biosensing.

## Conflicts of Interest

The authors declare no conflict of interest.

## Data Availability

The data described in the article are available at  https://zenodo.org/uploads/19452735 and https://zenodo.org/uploads/19451978. We would appreciate it if other researchers could benefit from our literature and results. This will foster discussions and collaboration among scientists worldwide
